# Microtubules play a role in trafficking prevacuolar compartments to vacuoles in tobacco pollen tubes

**DOI:** 10.1098/rsob.180078

**Published:** 2018-10-31

**Authors:** Elisabetta Onelli, Monica Scali, Marco Caccianiga, Nadia Stroppa, Piero Morandini, Giulio Pavesi, Alessandra Moscatelli

**Affiliations:** 1 Department of Biosciences, Milan University, Via Celoria 26, 20133 Milan, Italy; 2 Department of Life Science, Siena University, Via A. Moro 2, 53100 Siena, Italy

**Keywords:** microtubules, prevacuolar compartment, pollen tube, *Nicotiana tabacum*, vacuole

## Abstract

Fine regulation of exocytosis and endocytosis plays a basic role in pollen tube growth. Excess plasma membrane secreted during pollen tube elongation is known to be retrieved by endocytosis and partially reused in secretory pathways through the Golgi apparatus. Dissection of endocytosis has enabled distinct degradation pathways to be identified in tobacco pollen tubes and has shown that microtubules influence the transport of plasma membrane internalized in the tip region to vacuoles. Here, we used different drugs affecting the polymerization state of microtubules together with SYP21, a marker of prevacuolar compartments, to characterize trafficking of prevacuolar compartments in *Nicotiana tabacum* pollen tubes. Ultrastructural and biochemical analysis showed that microtubules bind SYP21-positive microsomes. Transient transformation of pollen tubes with LAT52-YFP-SYP21 revealed that microtubules play a key role in the delivery of prevacuolar compartments to tubular vacuoles.

## 1. Introduction

Pollen tubes are tip-growing cells that convey sperm to the embryo sac for double fertilization in angiosperms [1,2]. However, they are also intriguing cell models for studying membrane trafficking during polarized cell growth. Continuous plasma membrane (PM) recycling maintains distinct functional domains in the apex (5 µm from the tip PM) with respect to the shank (5–25 µm from the tip PM) and distal region (behind the male germ unit), and is due to a fine balance between exocytosis and endocytosis [3,4].

Time-lapse analysis and ultrastructural observations using charged nanogold has partially characterized two endocytic pathways. Plasma membrane internalized in the shank is mostly sent to the Golgi apparatus to be reused in the secretion pathway and partly conveyed to vacuoles through the trans-Golgi network (TGN) [5]. On the other hand, PM internalized in the apex mostly goes to the degradation pathway bypassing the Golgi/TGN apparatus [5]. Specific drugs affecting actin filament (AF) and microtubule (MT) integrity have further defined these degradation pathways: PM internalized in the shank is delivered to multivesicular bodies (MVBs)/prevacuolar compartments (PVCs) and then to vacuoles in an AF-dependent way [6], while PM endocytosed in the tip is conveyed to vacuoles, bypassing the Golgi/TGN, in a MT-dependent manner [7].

The routes of vacuole delivery, which require dynamic interaction between membrane compartments and the cytoskeletal apparatus, have not been fully characterized in pollen tubes. In lily, PVCs were identified by the presence of LIVSR and BP80 and found to be distributed throughout the pollen tube, except in the apical inverted-cone region [8]. In addition, in *Arabidopsis thaliana* it was observed that vacuolar protein sorting 41 (AtVPS41) is involved in late events of degradation pathways in pollen tubes [9]. The importance of degradation pathways for proper pollen–pistil interaction was recently highlighted and the integrity of degradation pathways plays a crucial role in the proper transport of female cues to vacuoles, in vacuole biogenesis and in pollen tube penetration of style transmitting tissue [9].

It is largely accepted that AFs are responsible for the cytoplasmic streaming that transports organelles and vesicles in the plant cell cytoplasm [10]. In pollen tubes, long AF bundles convey secretory vesicles to the inverted cone region [10] where fine AFs organize into a cortical fringe that undergoes rapid turnover during pulsed growth [11]. The actin fringe plays a role in control of clear zone formation [12] and in exo/endocytosis in the apex and shank, being a prerequisite for pollen tube growth [6,13]. Given their key role in cytoplasmic streaming and pollen tube growth, the structure and function of AFs have been widely studied.

By contrast, the role of MTs in membrane trafficking needs to be characterized. In somatic cells, MTs take part in cell plate formation during cytokinesis and contribute to cell morphogenesis, regulating localized secretion of cellulose synthase complexes to the PM [14,15]. They also contribute to the fine positioning of organelles and are involved in determining organelle morphology and shaping [16–20]. In pollen tubes, MTs control movement of the male germ unit [21] and positioning of large vacuoles in the distal regions of the tube [22]. More recently, it was reported that MTs also play a role in exocytosis in the central region of the tip and in endosome trafficking [7,19]. Specifically, MT perturbation by nocodazole delayed transport of endocytic vesicles to the vacuoles [7] and redirected the endocytosed material to the Golgi apparatus, suggesting that MTs are involved in transport of endosomes towards vacuoles [7].

As the putative function of MTs in degradation pathways has not yet been thoroughly investigated in pollen tubes, the goal of this study is to characterize membrane trafficking to vacuoles and the role of MTs in these pathways.

For this purpose, different drugs affecting MT polymerization were employed together with SYP21 as a marker of PVCs [23–25]. Binding experiments using taxol-purified MTs and biochemical analysis revealed that MTs interact with SYP21-positive compartments *in vitro*. Transient transformation of pollen grains with pLAT52:YFP-SYP21 plasmid showed that SYP21 localized in round organelles identified as PVCs and on tubular vacuoles in growing tobacco pollen tubes. Perturbation of MTs by different drugs revealed that they are involved in endosome trafficking and in mediating PVC delivery to, and/or fusion with, tubular vacuoles.

## 2. Results

### 2.1. Microtubules preferentially bind organelles involved in degradation pathways

In a previous study, internalization assays using charged nanogold showed that MTs play a role in the movement of endocytic vesicles, internalized in the clear zone, towards vacuoles [7].

**Figure 1.**
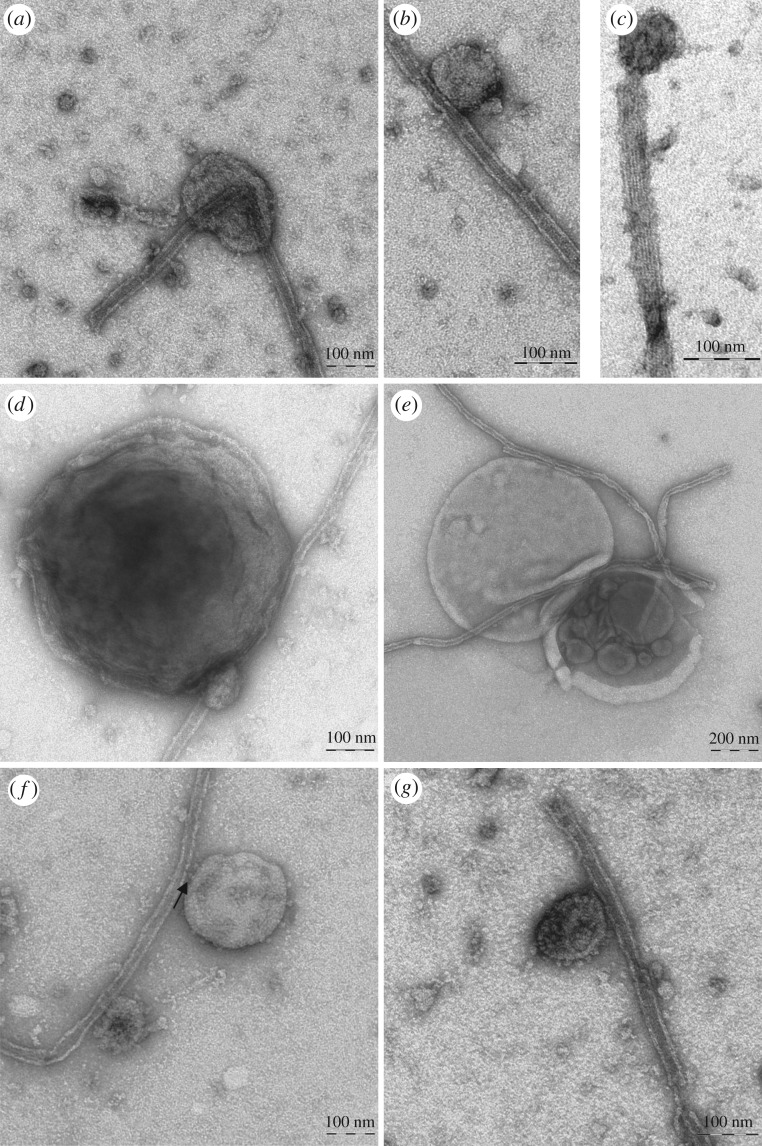
Taxol-purified MTs bind pollen tube microsomes *in vitro.* (*a–c*) Organelles bound to MTs show sizes ranging from 50 to 200 nm in diameter. These organelles, delimited by smooth membranes, preferentially bind to the MT wall and are only occasionally observed at MT ends (*c*). (*d,e*) MTs also bind compartments larger than 600 nm delimited by smooth membranes. Like MVBs, some of these compartments show inner vesicles (*e*). (*f,g*) In some cases, outer membranes of MT-bound compartments are decorated with small particles that appear to mediate their interaction with MTs (arrow). Scale bars, *a–d,f,g* = 100 nm; *e* = 200 nm.

To confirm that MTs interact with specific organelles, taxol-stabilized MTs were incubated with microsomes purified from tobacco pollen tubes in the presence of AMP-PNP, which allows ATP-dependent permanent binding between MTs and organelles. Transmission electron microscope (TEM) observations showed that about 40% of organelles observed on the grids interact with MTs (figure 1). Most of these compartments showed diameters from 50 to 500 nm (figure 1) and organelles larger than 600 nm were also observed (about 10%; figure 1*d,e*). The membrane delimiting most of these compartments appeared smooth and, as observed in MVBs, inner vesicles were evident (figure 1*e*). In other cases, the outer membrane was decorated with small particles that seem to mediate the interaction with MTs (figure 1*f* arrow, *g*). Binding of organelles to MTs occurred preferentially along the MT wall and only occasionally at their end (about 5%; figure 1*c*). The unbound organelles in the grids were isolated (electronic supplementary material, figure S1A,B), suggesting that binding with MTs was not due to high organelle concentration. In the absence of AMP-PNP very few organelles (about 5%) appeared associated with MTs, confirming that AMP-PNP stabilized organelles/MTs binding.

The binding experiments therefore showed that MTs interact with different membrane compartments in tobacco pollen tubes.

**Figure 2.**
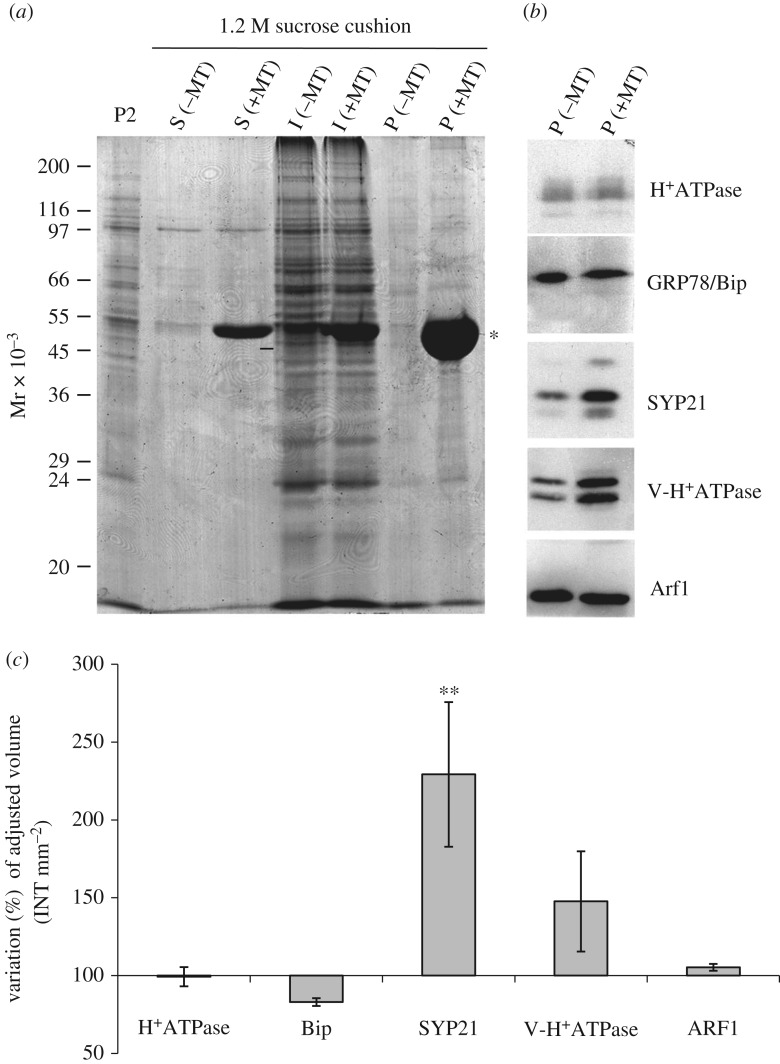
Biochemical characterization of MT-binding experiments. (*a*) Electrophoretic profile of pollen tube microsomal pellet (P) sedimented through a sucrose cushion in the presence or absence of taxol-stabilized MTs (+/–MT, respectively). S lanes show the electrophoretic pattern of soluble polypeptides that do not enter the sucrose cushion irrespective of the presence (+MT) or absence of MTs (–MT). Most organelles remain at the cushion interface in control experiments (I −MT) and MT assays (I +MT). Tubulin was detected particularly in the P +MT fraction (asterisk). (*b*) Antibodies against H^+^ATPase, GRP78/Bip, Arf1, V-H^+^ATPase and SYP21, probed by western blot on P +/–MT samples, recognize protein markers for PM, ER, Golgi apparatus, vacuoles and PVCs. (*c*) Quantitative analysis using Quantity One software shows significant (Student's *t*-test, **p < 0.01) enrichment of SYP21 in P +MT compared to P –MT samples. The graph shows adjusted volume (intensity (INT) mm^−2^) and percentage variation in P +MT with respect to P –MT samples after normalization to the latter. Enrichment of V-H^+^ATPase was not significant (Student's *t*-test, *p* > 0.05). Error bar indicates standard error (*n* = 4).

To further confirm the interaction between MTs and organelles and in order to investigate the identity of these compartments, western blot analysis was performed (figure 2). Microsomes, incubated with or without MTs (+ or –MT, respectively), were collected by centrifuging through 1.2 M sucrose cushions. The cushion made it possible to separate MT-bound organelles, recovered in the pellet (P fraction), from free organelles, which mostly remained on the surface of the cushion (I fraction). Electrophoretic analysis showed that most proteins were recovered in the I fraction, while the solubilized proteins (S) and P fractions had a lower protein content (figure 2*a*), suggesting that most unbound organelles remained on the cushion. Tubulin was detected in the P fraction, as expected (compare +MT and –MT in figure 2*a*; asterisk). The P fractions with and without MTs (P +/–MT, respectively) were probed using antibodies against organelle-specific markers (figure 2*b*). Western blot and quantitative analysis performed in four independent experiments showed significant enrichment of SYP21 in P +MT compared to P –MT samples (figure 2*b,c*), suggesting that MTs interact preferentially with organelles involved in the late degradation pathway. Although enrichment of V-H^+^ATPase was observed in P +MT, it was not statistically significant (figure 2*c*).

Antibodies against H^+^ATPase, GRP78/Bip and Arf1, which recognize protein markers of PM, endoplasmic reticulum (ER) and Golgi apparatus, respectively [26–28], did not reveal any difference in P +MT and P –MT samples (figure 2*b,c*), suggesting that these organelles partly pelleted through the sucrose cushion independently of MTs.

**Figure 3.**
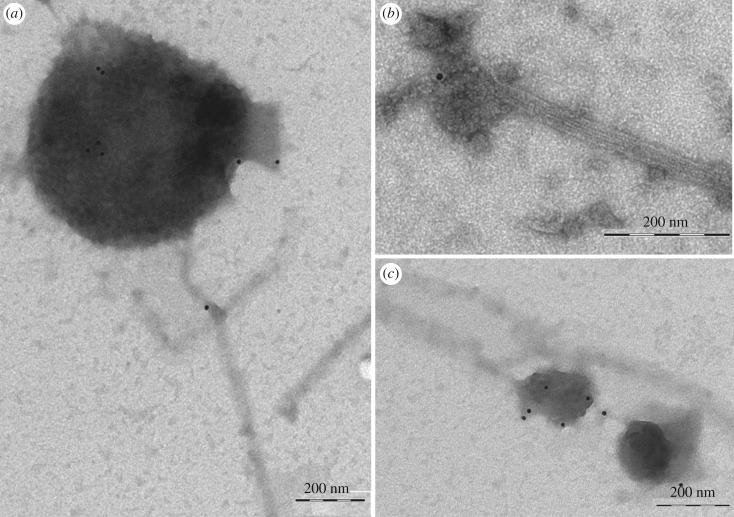
Immunogold labelling of MT-bound compartments. (*a–c*) Anti-SYP21 antibody labels organelles bound to taxol-stabilized MTs. The diameter of labelled compartments ranges from 100 to 700 nm. Scale bar, *a–c* = 200 nm.

The antibody against SYP21 was also used in immunogold studies in MT-bound organelles (figure 3). About 40% of organelles observed in the grids were labelled by SYP21 antibody in four different experiments (50 images for each experiment were considered). The diameter of labelled compartments ranged from 50 nm to 600 nm (figure 3) in line with the size reported for PVCs in somatic cells [24]. Control experiments performed with normal rabbit serum and with secondary antibody did not show any staining (electronic supplementary material, figure S1C,D, respectively).

These results further sustain the hypothesis that MTs preferentially bind compartments involved in degradation pathways in the tobacco pollen tube.

### 2.2. The binding between MTs and SYP21-positive organelles was specific and ATP-dependent

**Figure 4.**
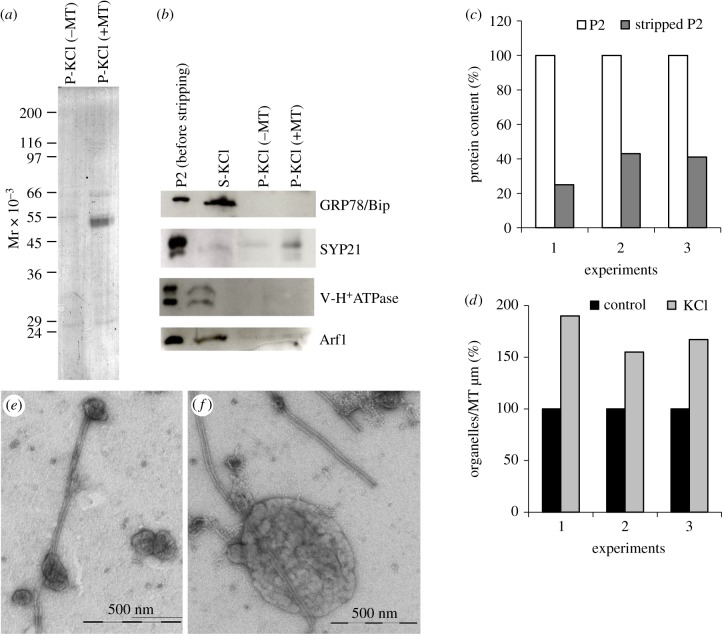
Binding specificity of pollen tube organelles to MTs. (*a*) Electrophoretic profiles of P fractions (+/–MT) after organelle stripping with KCl. (*b*) Western blot analysis of P samples +/–MT shows that GRP78/Bip, Arf1 and V-H^+^ATPase are recovered in the soluble fraction after KCl treatment, while SYP21 organelles are pelleted preferentially in +MT samples. (*c*) The graph shows the variation in percentage protein content after normalization to the unstripped P2 in three different experiments. The protein content of microsomes decreases after incubation with KCl. (*d*) The graph shows the percentage variation in organelle number per micrometre of MT in KCl-treated samples with respect to untreated microsomes, normalized to unstripped P2 in three independent experiments. Quantitative analysis of MT-bound KCl-stripped organelles shows that the number of compartments bound to MTs increased considerably with respect to untreated microsomes. (*e,f*) Ultrastructural observations show that the membrane delimiting KCl-treated organelles is smooth. Scale bars, *e,f* = 500 nm.

Enrichment of SYP21 in the P +MT fraction in the presence of AMP-PNP suggested a specific interaction. To confirm the specificity of the MT/SYP21 organelle binding, proteins on the surface of microsomes were stripped with a high concentration of KCl before incubation with MTs (figure 4). The efficiency of stripping was confirmed by a decrease in microsome protein content after KCl treatment (50% with respect to untreated organelles), in three different experiments (figure 4*c*). Western blot analysis using antibodies against protein markers of Golgi apparatus, ER and vacuoles showed that Arf1, GRP78/Bip and the epsilon subunit of V-H^+^ATPase were stripped from the organelle surface and recovered in the soluble fraction after salt treatment (figure 4*a*, SDS-PAGE; figure 4*b*, western blot, S-KCl lane). Conversely, SYP21 co-pelleted with MTs as expected, since it has a transmembrane domain in the C-terminus (figure 4*b*, P-KCl −/+MT lane) [26].

Enrichment of SYP21 in the P +MT fraction could suggest that binding of SYP21-positive organelles may not be mediated by surface proteins. However, TEM analysis of MT-bound organelles after KCl stripping showed that the number of compartments bound to MTs increased considerably with respect to untreated microsomes in three different experiments (figure 4*d*) and the organelle delimiting membranes appeared smoother than those of untreated microsomes (figure 4*e,f*; comparison of figure 1).

These findings suggested that specific organelle surface proteins could regulate the interaction with MTs, since in their absence random interactions between MTs and organelles were observed.

**Figure 5.**
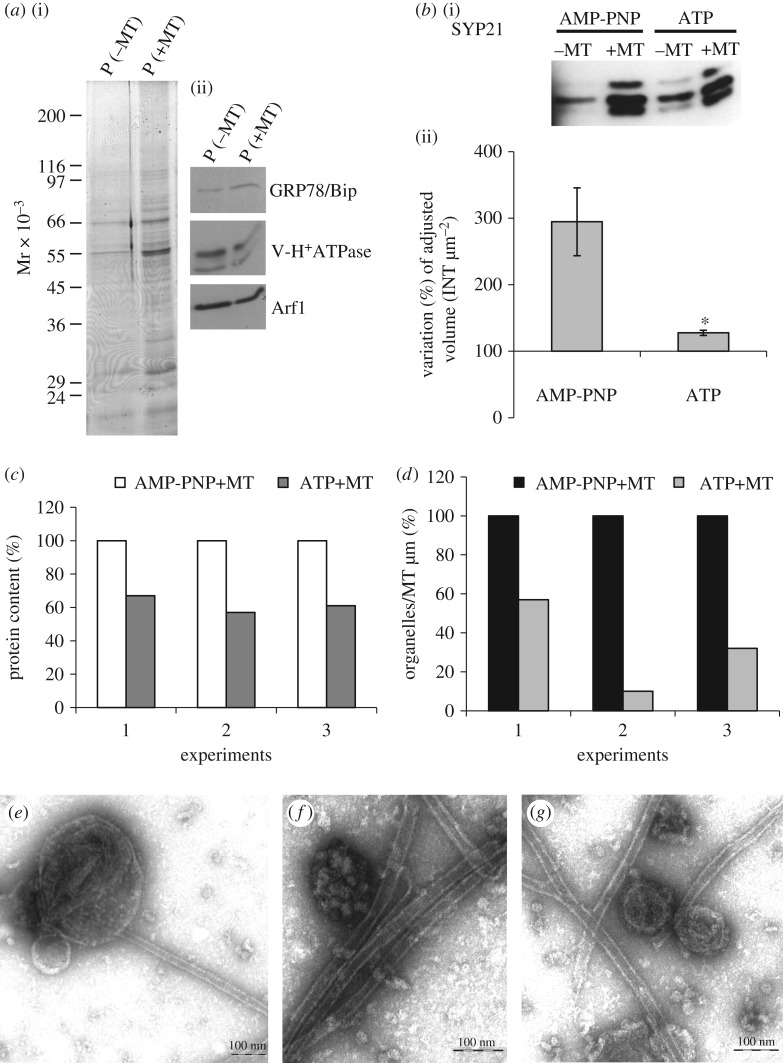
Microsomes bind MTs in an ATP-dependent manner. (*a*) Electrophoretic profiles of P +/–MT fractions in the presence of ATP (i) and western blot analysis using GRP78/Bip, V-H^+^ ATPase, SYP 21 and Arf1 antibodies on P +/–MT fractions in the presence of ATP (ii). In the presence of ATP, the same content of ER, vacuole and Golgi markers is detected in –MT and +MT samples. (*b*) Western blot experiments using anti-SYP21 antibody show that SYP21-positive compartments are more frequent in P +MT than in P –MT samples in the presence of AMP-PNP and ATP (i). Quantitative analysis using Quantity One software (ii) shows percentage variation in adjusted volume of +MT with respect to –MT (assumed 100%) after incubation with ATP or AMP-PNP (mean values; Student's *t*-test, *p < 0.05; error bar indicates standard error; *n* = 4). Fewer SYP21-positive organelles bound to MTs in the presence of ATP than in the presence of AMP-PNP. (*c*) The graph shows the variation in percentage protein content after normalization to AMP-PNP-treated P2 in three different experiments. In the presence of ATP, less protein is recovered in the P fractions. (*d*) The graph shows the percentage variation in organelle number per micrometre of MT in ATP-treated samples with respect to AMP-PNP treated microsomes, normalized to AMP-PNP samples in three independent experiments Quantitative analysis shows fewer organelles per micrometre of MT in P +MT pellets of ATP-treated samples than in samples incubated with AMP-PNP. (*e–g*) Ultrastructural observations do not show differences in the morphology of MT-bound organelles between ATP- and AMP-PNP-treated samples. Scale bar, *e–g* = 100 nm.

The use of AMP-PNP suggested that proteins having ATPase activity could be responsible for the binding between SYP21 organelles and MTs. To investigate the nature of this interaction in more detail, binding experiments were performed in the presence of ATP instead of AMP-PNP (figure 5). Western blot analysis showed that in the presence of ATP, SYP21 was still enriched in the P +MT with respect to the P –MT fraction (figure 5*b*, comparison of −/+ MT), providing further support for binding specificity of SYP21 organelles to MTs. However, the amount of SYP21 in the +MT pellet in the presence of ATP, calculated in four different experiments, was considerably lower than in P +MT in the presence of AMP-PNP (figure 5*b*; graph), suggesting that the SYP21 compartments interact cyclically with MTs in an ATP-dependent manner. The cycling binding of SYP21 organelles with MTs was also confirmed by evidence that the amount of proteins in the MT pellet in the presence of ATP was considerably lower than in MT pellets in the presence of AMP-PNP (figure 5*c*). On the other hand, the amount of GRP78/Bip-, V-H^+^ ATPase- and Arf1-positive organelles did not change in P +MT in the presence of ATP (figure 5*a*, comparison of P −/+MT).

Moreover, TEM analysis of MT pellets obtained in the presence of ATP showed that the morphology of organelles bound to MTs was similar to that observed in the presence of AMP-PNP (figure 5*e–g*; comparison of figure 1) and confirmed that the number of organelles bound to MTs decreased with respect to experiments using AMP-PNP (figure 5*d*).

All these experiments further support the idea that the interaction of SYP21 organelles with MTs may be mediated by ATP-dependent proteins. However, additional experiments are necessary to identify and characterize the proteins mediating the binding between SYP21-positive compartments and MTs.

### 2.3. Sub-fractionation experiments showed a relationship between MTs and organelles involved in the degradation pathway

**Figure 6.**
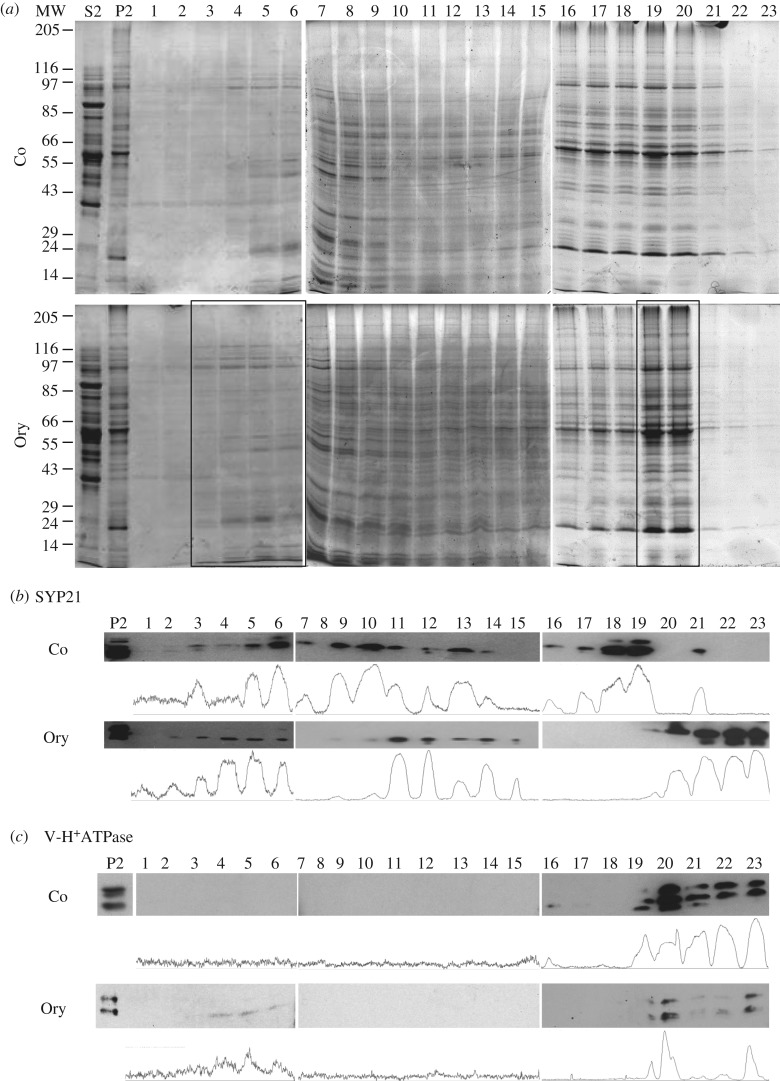
MT depolymerization by oryzalin affects migration of SYP21-positive compartments in sub-fractionation experiments. (*a*) Electrophoretic profiles of microsomes separated through continuous sucrose gradients. Microsome fractions of control pollen tubes (Co; upper panel) compared to those of pollen tubes grown in the presence of oryzalin (Ory; lower panel). A wider distribution of polypeptides having molecular mass between 150 and 97 kDa is evident in the oryzalin sample (fractions 2–6, box) than in control (fractions 4–6). In contrast, polypeptides in control fractions 16–23 are widely distributed with respect to oryzalin-treated samples which peaked in fractions 19–20 (box). (*b*) Western blot analysis using anti-SYP21 antibody in control pollen tubes continuously detected SYP21 in fractions 3–19 with peaks in fractions 6, 10, 13, 18–19 and 21 (see also ImageJ quantification analysis). Oryzalin treatment alters the distribution of SYP21-positive compartments, since SYP21 is present in two peaks (fractions 2–6 and 11–15) and in fractions 19–23. (*c*) Anti V-H^+^ATPase antibodies mark compartments in fractions 19–23 in control and oryzalin-treated pollen tubes. After oryzalin treatment, the compartments recognized by the V-H^+^ATPase antibody are also recorded in the lower density fractions 4–6.

To better define the role of MTs in trafficking towards vacuoles, we analysed the effect of low concentration of oryzalin (a drug which dramatically depolymerizes MTs in pollen tubes) [7,22] on the distribution of membrane compartments after centrifuging through a continuous sucrose density gradient, using antibodies against SYP21 and V-H^+^ATPase (figure 6). To minimize the side effects of oryzalin on pollen tube growth, we used a concentration of the drug (0.1 µM) that did not induce loss of cytoplasmic polarity or descent of big vacuoles into the apex (see §2.5).

Electrophoretic analysis of sucrose density fractions, derived from pollen tubes grown with or without oryzalin, showed greater modifications of polypeptide pattern in fractions 3–7 and 16–21 (figure 6*a*). Specifically, polypeptides with molecular mass in the range 97–150 kDa showed a wider distribution in oryzalin-treated samples (fractions 2–6, see box in figure 6*a*) than in controls (fractions 4–6). On the other hand, polypeptides that were widely distributed in the control experiments (fractions 16–23) were present in fractions 16–20 and peaked in fractions 19–20 in oryzalin-treated samples (see box in figure 6*a*).

These experiments showed that oryzalin altered the mobility of membrane compartments through the sucrose density gradient, suggesting that MT depolymerization could affect membrane trafficking, leading to changes in membrane compartment composition.

To evaluate the effect of oryzalin on the distribution of compartments involved in late degradation pathways, the fractions obtained after centrifuging microsomes through sucrose density gradients were probed using anti-SYP21 and anti-V-H^+^ATPase antibodies as markers of PVCs and vacuoles, respectively (figure 6*b,c*). In immunoblot assays of control pollen tubes, SYP21 was detected continuously in fractions 3–14 with three distinct peaks in fractions 6, 10 and 13. It was also present in fractions 16–19, 21 (figure 6*b*). Otherwise, in sub-fractionation experiments, SYP21 was only present in two distinct peaks in fractions 2–6 and 11–15 of oryzalin-treated pollen tubes. SYP21 was also observed in the higher sucrose density fractions 19–23.

In western blot experiments, anti-V-H^+^ATPase labelled compartments in high-density fractions 20–23 of control pollen tubes (figure 6*c*), whereas after oryzalin treatment the membrane compartments recognized by V-H^+^ATPase antibody were also recorded in fractions 4–6 (figure 6*c*). Notably, V-H^+^ATPase- and SYP21-labelled organelles partially overlapped in the same fraction (19, 21) in control pollen tubes, while after oryzalin treatment this overlap was enhanced both in high- (20–23) and low-density fractions (4–6), suggesting that the membrane composition and content of these organelles were altered by MT depolymerization.

These observations suggest that MT depolymerization affected trafficking involving both the SYP21 and V-H^+^ATPase compartments.

MT depolymerization also affected Bip1 and Arf1 distribution throughout the sucrose density gradient in different ways (electronic supplementary material, figure S2). In control pollen-tube-derived microsomes, Bip1 was detected in low (3–7; electronic supplementary material, figure S2A) and high sucrose density fractions (20–23; electronic supplementary material, figure S2A). Oryzalin treatment maintained this distribution, also spreading Bip1 in fractions 1 and 2. Arf1 was widely distributed through the sucrose density gradient in control experiments (electronic supplementary material, figure S2B), while MT depolymerization restricted its distribution (3, 10–11, 15, 18; electronic supplementary material, figure S2B) and decreased its presence particularly in the higher sucrose density fractions.

### 2.4. SYP21 is localized on PVCs and tubular vacuoles in tobacco pollen tubes

**Figure 7.**
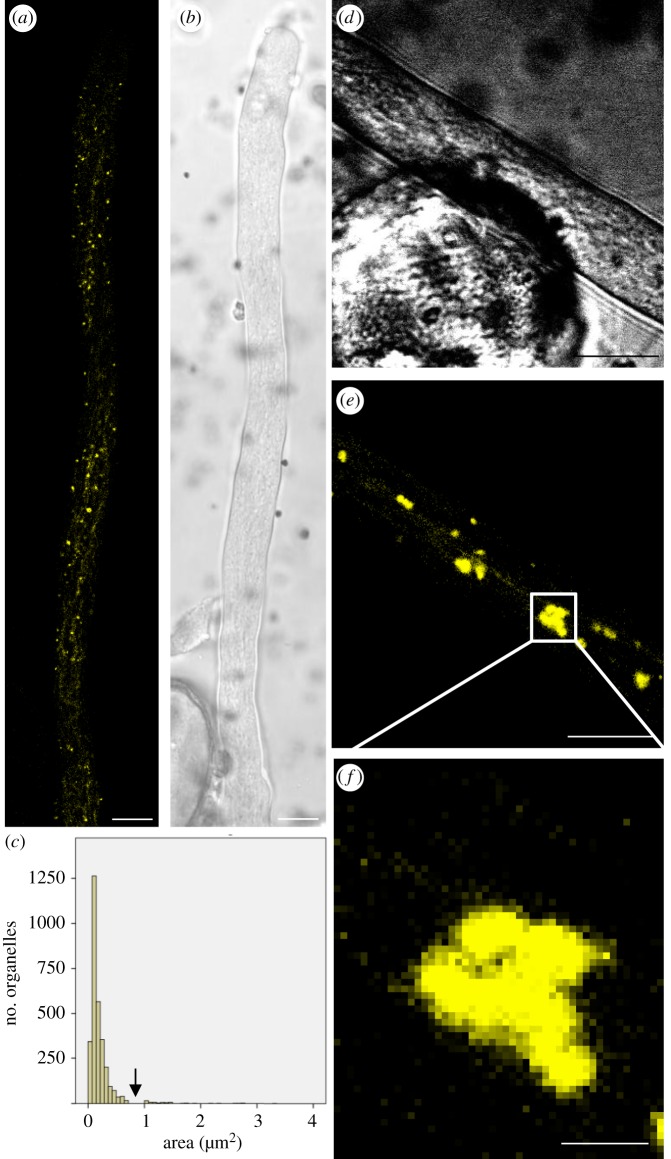
Identification of PVCs by transient transformation of pollen tubes with pLAT52:YFP-SYP21. (*a,b*) YFP-SYP21 detects round organelles widely distributed in pollen tubes except in the tip and more distal regions where there are large vacuoles. YFP-SYP21 also localizes in dynamic tubular compartments spanning the pollen tube longitudinally (*b*, bright field). (*c*) Round organelles have a mean area of about 0.2 µm^2^. Most SYP21-positive compartments (98%) measure between 0.05 and 0.7 µm^2^ with a peak at 0.1–0.2 µm^2^. A discontinuity is evident in the frequency distribution of SYP21-positive organelle area between 0.7 and 1.0 µm^2^ (arrow). (*d–f*) Overexpression of YFP-SYP21 induces aggregation of round organelles into large clusters (*d*, bright field). Scale bar, *a–e* = 10 µm; *f* = 2.5 µm.

To study the localization of SYP21 in growing pollen tubes, pollen grains were transiently transformed with YFP-SYP21 construct under the control of pollen-specific promoter LAT52. We tested growing plasmid concentrations and the lowest concentration that allows to observe SYP21 staining was chosen. Since we elicited ectopic expression of *Arabidopsis* SYP21 in *Nicotiana tabacum* pollen tubes, the average growth rate of untransformed cells was compared with that of pLAT52/YFP-SYP21-transformed pollen tubes in order to avoid undesired side-effects on pollen tube physiology: the transformation did not significantly affect pollen tube growth rate (Student's *t*-test, *p* *>* 0.05; electronic supplementary material, figure S2C). YFP-SYP21 revealed round organelles (figure 7*a,b*) having a mean area of 0.2 µm^2^ in transformed pollen tubes (figure 7*c*). The area of most of SYP21-labelled compartments (98%) was in the range 0.05–0.70 µm^2^ with a peak at 0.1–0.2 µm^2^ (figure 7*c*). Only 2% of SYP21-positive compartments exceeded 1 µm^2^, suggesting that these large bodies could be caused by exogenous expression of SYP21 which induced aggregation of organelles. Notably, a discontinuity in the frequency distribution was observed, since no compartments with areas between 0.7 and 1.0 µm^2^ were detected (figure 7*c*, arrow). These areas were coherent with SYP21 organelle size observed in immunogold analysis of binding experiments using SYP21 antibody (figure 3). In addition, TEM observation showed SYP21-positive organelles with a diameter of 50 nm, which were not revealed by laser confocal microscopy. SYP-21-positive organelles exhibited long-range movements along the pollen tube, excluding the clear zone, a transportation pattern typical of AF-dependent cytoplasmic streaming (electronic supplementary material, movie S1).

Overexpression of YFP-SYP21 in pollen tubes induced aggregation of positive organelles to form larger structures (figure 7*d,e*), which were clusters of smaller bodies (figure 7*f*). These clusters took several shapes and moved in an irregular way along the pollen tube, showing quick movements alternating with pauses (data not shown). SYP21-positive clusters never reached the apex of the pollen tube. Pollen tube growth was not affected by SYP21 overexpression (from 3.7 µm min^−1^ in the control to 3.4 µm min^−1^ in SYP21-overexpressed pollen tubes; Student's *t*-test, *p* *>* 0.05). Thus, overexpression induced clusters as observed in somatic cells [24], suggesting that SYP21-positive organelles in tobacco pollen tubes can also be identified as PVCs.

**Figure 8.**
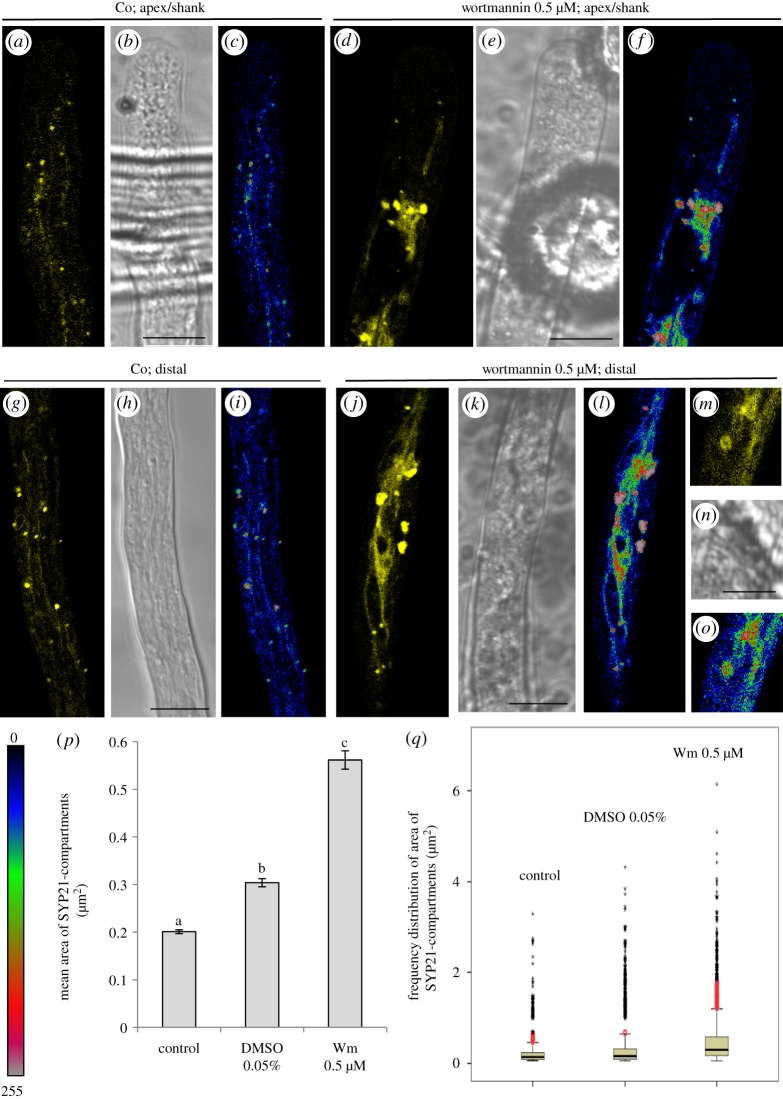
Wortmannin (Wm) treatment induces homotypic fusion of SYP21-positive compartments. (*a,b*) Distribution of YFP-SYP21 in the apex/shank of control pollen tubes (*b*, bright field). (*c*) Fluorescence intensity of YFP-SYP21 revealed by pseudo-coloured images (black/blue and red/grey indicated lower and higher fluorescence, respectively). (*d,e*) Distribution of YFP-SYP21 in the apex/shank of Wm-treated pollen tubes (*e*, bright field). (*f*) Fluorescence intensity of YFP-SYP21 revealed by pseudo-coloured images. (*g,h*) Distribution of YFP-SYP21 in the distal region of control pollen tubes. (*i*) Fluorescence intensity of YFP-SYP21 revealed by pseudo-coloured images. (*j,k*) Distribution of YFP-SYP21 in the distal region of Wm-treated pollen tubes. (*l*) Fluorescence intensity of YFP-SYP21 revealed by pseudo-coloured images. (*m,n*) Detail of YFP-SYP21 labelling on the surface of PVCs, induced by Wm treatment. (*o*) Fluorescence intensity of YFP-SYP21 revealed by pseudo-coloured images. All the pictures show growing pollen tubes. *a*–*l* Scale bar, 10 µm. (*p*) Graph shows mean area of SYP21-positive compartments in control, DMSO- and Wm-treated pollen tubes. A significant increase (*p* < 0.0001) in mean area of SYP21-positive round bodies was observed in Wm-treated with respect to control and DMSO-treated pollen tubes. Error bar indicates standard error. (*q*) Box plots of frequency distribution of areas of SYP21-positive bodies. Boxes show the interquartile range (Hspread) and median (horizontal line). The lines extend from the lowest to the highest value in 1.5 Hspread from the end of the box. Red circles represent values between 1.5 and 3 Hspread and black asterisks represent extreme values above 3 Hspread. The increase in mean area (see *m*) of round organelles after Wm treatment was due to an increase in the number of organelles having an area >1 µm^2^ and to an increase in PVC size (up to 6 µm^2^).

To obtain further evidence that SYP21-positive compartments are actually PVCs, the pollen tubes were treated with 0.5 µM wortmannin (Wm) (figure 8), which has been shown to affect PVC size in somatic cells [8,29,30]. This concentration of Wm did not significantly decrease pollen tube growth rate (Student's *t*-test, *p* > 0.05 with respect SYP21-transformed and 0.05% DMSO treated pollen tubes; electronic supplementary material, figure S2C) although about 40% of tubes did not grow. Wortmannin induced significant enlargement of SYP21-positive compartments (ANOVA test: *F*_6.11498_ = 140,439, *p* < 0.0001) with respect to controls (Tukey's test between control and Wm samples, *p* < 0.01) and 0.05% DMSO-treated pollen tubes (Tukey's test between DMSO and Wm samples, *p* < 0.01; figure 8*a–p*). The SYP21 labelling, clearly visible in the delimiting membrane, shows that Wm induced the fusion of SYP21-positive compartments, as expected (figure 8*m–o*), further sustaining that SYP21 identified PVCs in pollen tubes. Moreover, control and DMSO-treated pollen tubes were also significantly different (Tukey's test between control and DMSO samples, *p* < 0.01). In addition, areas reached 6 µm^2^ in the presence of Wm but did not exceed 4 µm^2^ in DMSO-control samples (figure 8*q*), and in controls and DMSO-treated pollen tubes the percentage of SYP21-positive organelles having an area greater than 1 µm^2^ was only 2% and 7%, respectively, while in Wm-treated pollen tubes it reached 20% (figure 8*q*).

To further confirm that SYP21-positive compartments were actually PVCs, tobacco pollen grains were transiently transformed with pLAT52/GFP-BP80-CT [8] (electronic supplementary material, figure S3). BP80 transformation did not affect pollen tube growth rate (from 4.3 µm min^−1^ in control to 4.1 µm min^−1^ in BP80 pollen tubes; Student's *t*-test, *p* *>* 0.05). Like with SYP21, BP80-positive compartments appeared as rounded organelles distributed in the pollen tube cytoplasm, excluding the apex (electronic supplementary material, figure S3A–D). To ensure that SYP21 and BP80 stained the same particles, cotransformation experiments using pLAT52/YFP-SYP21 and pLAT52/GFP-BP80 were performed (electronic supplementary material, figure S3F–M). To avoid the interference of YFP and GFP signals, transformed pollen tubes were observed as reported by Foresti *et al.* [24]. Crosstalk experiments showed that the emission spectrum of YFP and GFP does not overlap (electronic supplementary material, figure S4A–F). Both in the apex/shank and in the distal region, round organelles appeared simultaneously stained with YFP-SYP21 and GFP-BP80 (electronic supplementary material, figure S3F–M). The cotransformation induced a lower signal in both channels with respect to single transformation. Moreover, to further sustain the presence of YFP-SYP21 and GFP-BP80 on the same organelles, the frequency distribution of BP80-positive organelle areas were analysed and compared with that of SYP21-positive compartments: the area distribution of two kind of organelles perfectly overlapped in the 0.05–0.7 µm^2^ range (electronic supplementary material, figure S3E). The Pearson correlation of the two distributions of the frequencies with which each value was detected by SYP21 and B80 was high (*r* = 0.95). Likewise, the Mann–Whitney paired test did not detect any significant difference between the two distributions (*p*-value > 0.05). In the graph (electronic supplementary material, figure S3E) only organelles with an area less than 0.7 µm^2^ were considered, since the largest BP80-PVCs measured 0.63 µm^2^. Together this data revealed that BP80 and SYP21 both identify PVCs.

Besides localizing in round organelles, surprisingly SYP21 also seemed to localize along tubular structures spanning the pollen tube longitudinally (figure 7*a*). These structures looked like the dynamic tubular compartments already observed in pollen tubes [7,31]. The compartments were distributed in the shank and distal regions of the tube but did not reach the tip (figure 8*a,b,g,h*). On the contrary, BP80 did not show this longitudinal distribution (electronic supplementary material, figure S3A,C).

Control transformation experiments using pLAT52/YFP did not significantly affect pollen tube growth (from 4.4 µm min^−1^ in the untransformed to 5.4 µm min^−1^ in YFP pollen tubes; Student's *t*-test, *p* > 0.05) and showed a diffuse cytoplasmic staining suggesting that YFP, differently from pLAT52/YFP-SYP21, does not have a specific localization (electronic supplementary material, figure S5A–F).

Interestingly, these SYP21-positive tubular compartments were affected by Wm, which induced tubule aggregation (figure 8*d–f, j–o*), suggesting that Wm could have an effect similar to that observed in round organelles. In addition, Wm treatment increased the localization of SYP21 on the tubular compartments in the shank and distal regions, with respect to controls (figure 8, compare *a*–*c* with *d*–*f* and *g*–*i* with *j*–*o*). Notably, since low concentrations of Wm that promote homotypic fusion of PVCs did not cause aggregation between round and tubular compartments, we may suppose that they are functionally distinct compartments.

In pollen tubes, Wm (0.8 µM) has been reported to affect AFs [30], whereas its influence on MTs has not been investigated. To ascertain that the homotypic fusion of PVCs and tubular vacuole coalescence described above were not side-effects of cytoskeletal alterations, the distribution pattern of AFs in pollen tubes incubated with 0.5 µM Wm was studied by transient transformation with pLAT52/Lifeact-mEGFP (electronic supplementary material, figure S6). In growing pollen tubes, no differences were observed in the organization of the actin fringe or of AFs in the shank and distal regions, among controls, Wm- and 0.05% DMSO-treated cells (electronic supplementary material, figure S6A–C).

When the effect of a low concentration of Wm on MTs was investigated by immunofluorescence microscopy, it seemed to have a stabilizing effect in tobacco pollen tubes, since long MT bundles were observed in the shank (electronic supplementary material, figure S7G), whereas only short randomly oriented MT strands were observed in controls and 0.05% DMSO-treated pollen tubes (electronic supplementary material, figure S7A,B).

The tubular compartments stained by YFP-SYP21 resembled tubular vacuoles previously identified in pollen tubes by CDFDA [7,31]. To unambiguously identify the SYP21-positive tubular compartments, pollen tubes were stained with yeast vacuole marker Blue-CMAC, henceforth CMAC (electronic supplementary material, figure S5G–L) [32,33]. This dye is a membrane-permeable chloromethyl coumarin derivative that is largely sequestered into vacuoles. The network of interconnected tubules stained by CMAC extended longitudinally in the cell from the shank (about 5 µm from the apical PM) to the distal area (electronic supplementary material, figure S5G–L). These tubules were highly dynamic and sometimes seemed to outline the V-shaped apical domain (electronic supplementary material, figure S5G,H; see box). Large vacuoles in the oldest parts of the tubes were also stained by CMAC and appeared to arise by coalescence of tubular vacuoles (electronic supplementary material, figure S5 K–L; see box).

In order to exclude that transient transformation experiments induced changes in morphology and behaviour of membrane compartments involved in the degradative pathways, the simultaneous staining with CMAC of pLAT52/YFP transient transformed pollen tubes showed that morphology and distribution of tubular vacuoles were not affected (electronic supplementary material, figure S5E,F). Further attempts to visualized PVCs by using acridine orange in pLAT52/YFP transformed pollen tubes failed because the pollen tubes died or burst (data not shown).

**Figure 9.**
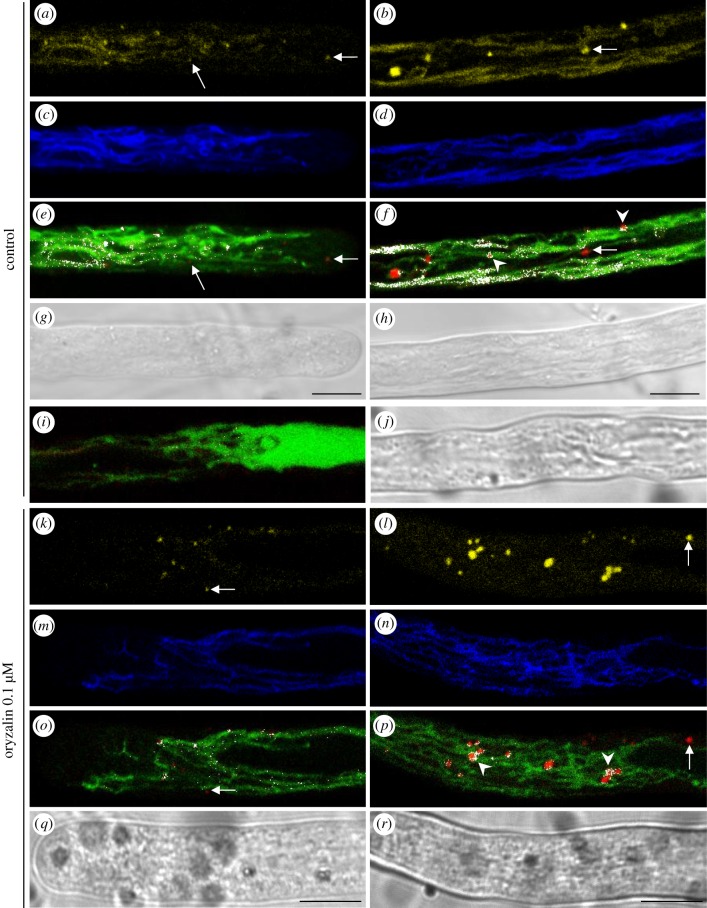
MT depolymerization by oryzalin affects PVC maturation and delivery to and/or fusion with tubular vacuoles. (*a–j*) YFP-SYP21/CMAC colocalization experiments in the apex/shank and distal areas of control pollen tubes. Yellow staining indicates SYP21 localized on round bodies and tubular compartments in the apex/shank and distal area (*a* and *b*, respectively). Blue staining of tubular compartments by CMAC reveals that they are indeed tubular vacuoles (*c* apex/shank, *d* distal area). White spots represent SYP21/CMAC colocalization points (*e* apex/shank, *f* distal area). In control pollen tubes, YFP-SYP21 colocalizes with CMAC on the delimiting membrane of PVCs (*f*; see arrowheads) and on tubular compartments. Distal vacuoles did show colocalization points (*i* and *j*). Bright field images of apical and distal regions of pollen tubes (*g* and *h*, respectively). (*k–r*) YFP-SYP21/CMAC colocalization experiments in the apex and distal area of oryzalin-treated pollen tubes. Yellow staining represents YFP-SYP21 distribution in the apical and distal regions (*k* and *l*, respectively). Blue staining shows tubular vacuoles stained with CMAC (*m* apex/shank, *n* distal area). SYP21/CMAC colocalization is shown as white points (*m* apex/shank, *n* distal area). After oryzalin treatment, colocalization points concentrate on PVCs and disappear almost completely from tubular vacuoles (*m* and *n*). Loss of SYP21 in tubular vacuoles is greater in the distal area than in the apex (*k–r*). The colocalization experiments show an absence of colocalization (*o* and *p*) of CMAC and SYP21 in several PVCs in control and treated pollen tubes (see arrows). Bright field images of apical and distal regions of pollen tubes (*q* and *r*, respectively). All the pictures are of growing pollen tubes. Scale bar, 10 µm.

**Figure 10.**
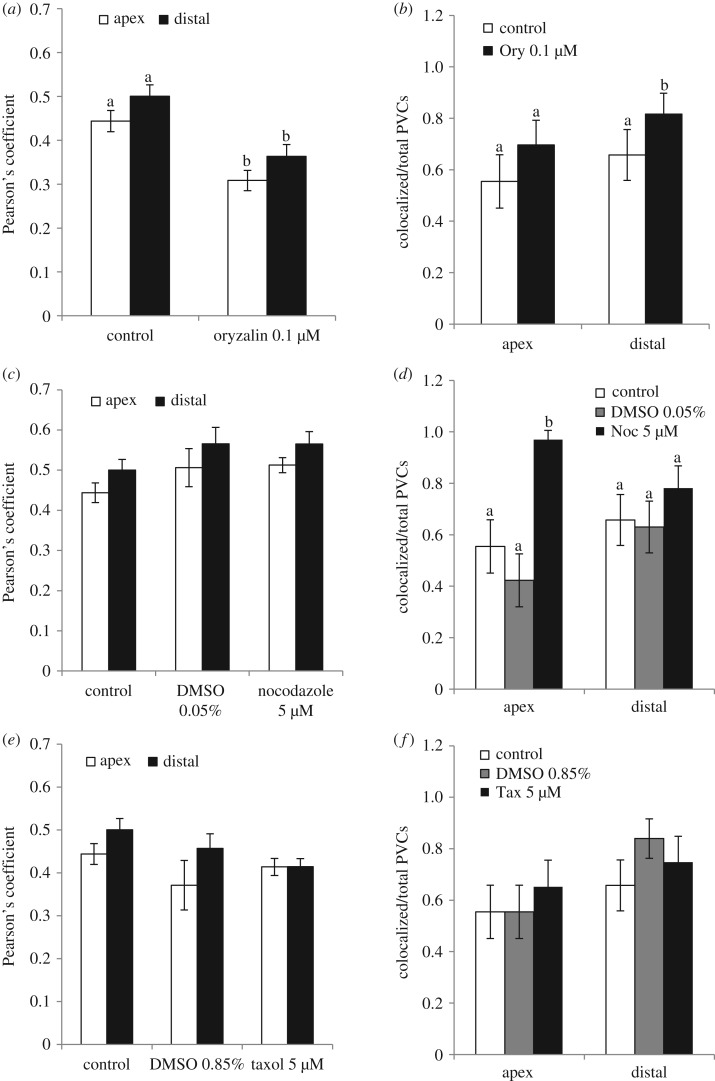
Colocalization experiments in the presence of MT drugs. (*a*) Pearson's coefficient shows a significant decrease in YFP-SYP21/CMAC colocalization in oryzalin-treated pollen tubes with respect to control (*p* < 0.01). (*b*) Number of PVCs colocalizing with CMAC with respect to the total number of PVCs observed in control and oryzalin (Ory)-treated pollen tubes. With oryzalin treatment, the number of PVCs showing YFP-SYP21/CMAC colocalization increases significantly in the distal area (*p* < 0.05). (*c*) Pearson's coefficient shows that nocodazole induces an increase in colocalization compared to control and 0.05% DMSO-treated pollen tubes. (*d*) Number of PVCs showing YFP-SYP21/CMAC colocalization in control, 0.05% DMSO- and 5 µM nocodazole (Noc)-treated pollen tubes. After nocodazole treatment an increase in colocalized PVCs with respect to total PVCs was observed in the apex/shank of pollen tubes (*p* < 0.01). (*e*) Pearson's coefficient shows that colocalization of YFP-SYP21 and CMAC in taxol-treated cells is not significantly different from colocalization in control and 0.85% DMSO-treated samples (*p* > 0.05). (*f*) Microtubule-stabilizing agents (taxol (Tax) and 0.85% DMSO) do not induce a significant increase in colocalized PVCs with respect to total PVCs (*p* > 0.05). The letters a and b indicate significantly different values. Error bar indicates standard error (*n* = 12).

In order to confirm the presence of SYP21 on tubular vacuoles, colocalization experiments were carried out, showing that YFP-SYP21 colocalized with CMAC on both PVCs and tubular compartments (figure 9*a–j*). The use of CMAC ensures that the emission spectrum does not overlap with that of YFP, as confirmed by crosstalk experiments (electronic supplementary material, figure S4G–L). No significant differences (Student's *t*-test, *p* > 0.05) in colocalization Pearson's coefficient were detected between the apical and distal regions in control pollen tubes (figure 10*a*). Interestingly, not all YFP-SYP21-positive PVCs colocalized with CMAC (figure 9*a–h*, arrows indicate PVCs not stained by CMAC; figure 10*b*). These data suggested the presence of free PVCs in the cytoplasm (not colocalizing with CMAC) and PVCs close to/fusing with tubular vacuoles (colocalizing with CMAC). Colocalization points were observed at the periphery of SYP21-positive vesicles, suggesting fusion events between PVCs and tubular vacuoles. Large vacuoles stained with CMAC were not labelled with YFP-SYP21 (electronic supplementary material, figure S8A,C,E,G).

To further characterize SYP21-positive organelles at the ultrastructural level, immunogold analysis was performed on pollen tubes (electronic supplementary material, figure S9; arrows). The anti-SYP21 antibody recognized compartments that were not uniform in morphology, appearing as vacuoles or small vesicles (electronic supplementary material, figure S9C), vesicle clusters (electronic supplementary material, figure S9D) or compartments comprising both vesicles and tubules (electronic supplementary material, figure S9B,E,F) and sometimes showing inner membranes (electronic supplementary material, figure S9E,F).

### 2.5. MT perturbation affected localization of SYP21 between PVCs and tubular vacuoles

To highlight the role of MTs in the degradation pathway involving SYP21-positive compartments, pollen tubes were treated with the MT depolymerizing agent oryzalin. Oryzalin treatment did not stop pollen tube growth, however it induced a significant decrease in growth rate compared to controls (Student's *t*-test, *p* < 0.01; electronic supplementary material, figure S2C).

In control tobacco pollen tubes, MTs are organized in longitudinal bundles in distal areas and in short, randomly oriented strands in the shank and apex (electronic supplementary material, figure S7A). Treatment with oryzalin depolymerized both the short MT strands and most distal bundled MTs (electronic supplementary material, figure S7D). To confirm a previous report showing that oryzalin did not affect AFs [7], tobacco pollen tubes transiently transformed with LAT52-lifeact and treated with oryzalin were observed (electronic supplementary material, figure S6D). No differences were revealed in the organization of the actin fringe or of AFs in the shank and distal regions, between oryzalin-treated pollen tubes and controls (electronic supplementary material, figure S6A,D). On the contrary, as already observed [34], pollen tube treatment with low concentration of LatB, which depolymerized AFs, induced a rearrangement of MTs in the apex/shank: the short MT fragments changed into long strands, often arranged in a helix structure along the long axis of the tube in the shank (electronic supplementary material, figure S7H) or organized in long filaments encapsulating the apex (electronic supplementary material, figure S7I).

**Figure 11.**
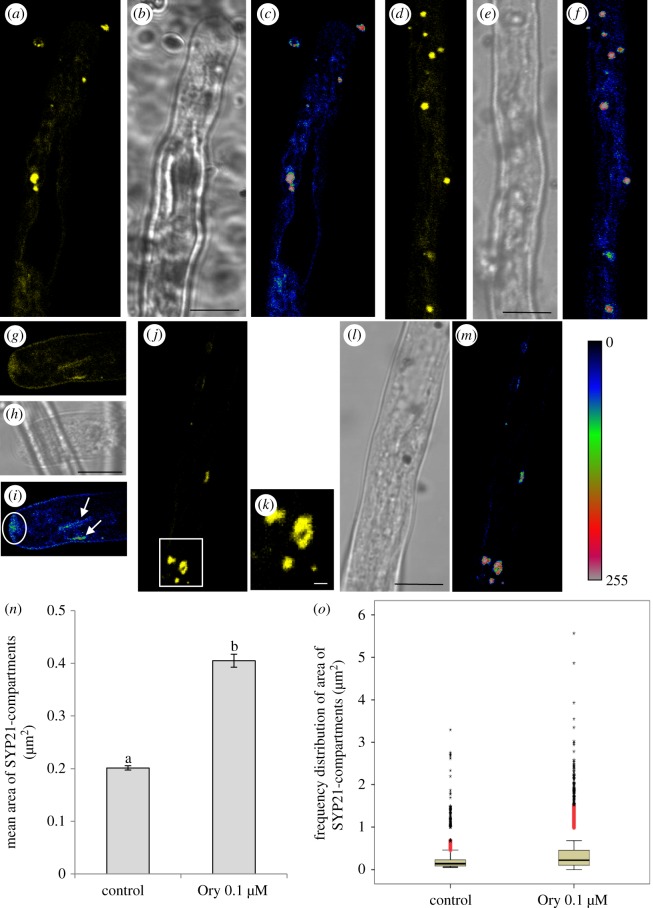
MT depolymerization by oryzalin affects trafficking of SYP21-positive compartments. (*a,b*) Oryzalin treatment enhances SYP21 staining on PVCs and considerably decreases the localization of SYP21 on tubular compartments in the apex/shank (*b*, bright field). (*c*) Pseudo-coloured images show YFP-SYP21 fluorescence intensity, which appears to be concentrated in PVCs. (*d,e*) The distribution of SYP21 in the distal area of oryzalin-treated pollen tubes is similar to that observed in the apex/shank (*e*, bright field). (*f*) Pseudo-coloured images show YFP-SYP21 fluorescence intensity which appears concentrated in PVCs. (*g–i*) SYP21 is occasionally found on the apical PM, on vesicles accumulating in the V-shape inverted cone region (*i*; circle; pseudo-coloured image) and on tubular compartments (*i*; arrows; pseudo-coloured image). (*j–l*) Labelling of YFP-SYP21 on the surface of large round PVCs (*l*, bright field). Magnification of PVC included in the box in (*j*) was shown in (*k*). (*m*) Pseudo-coloured images show YFP-SYP21 fluorescence intensity, which appears concentrated in PVCs and nearly lost in tubular vacuoles. All the pictures show growing pollen tubes. *a–j,l,m* scale bar, 10 µm; *k* scale bar, 1 µm. (*n*) The graph shows the mean area of SYP21 compartments in control and oryzalin-treated pollen tubes. The mean area of PVCs increased significantly compared with control (letters a and b indicate significantly different values, *p* < 0.0001). Error bar indicates standard error. (*o*) The box-plot shows that the number of PVCs having an area >1 µm^2^ increased considerably in oryzalin-treated cells with respect to control. Some PVCs reached 5 µm^2^ in area in the presence of oryzalin.

Microtubule depolymerization altered the localization of YFP-SYP21, which was only observed on round organelles (PVCs) and disappeared or decreased considerably on tubular compartments, in the apical and distal areas (compare figure 8*a–c, g–i* and figure 11*a–f, j–m*). In parallel with the decrease in YFP-SYP21 on tubular vacuoles, the mean area of PVCs increased significantly (0.4 µm^2^) compared with control (ANOVA test: *F*_6,11498_ = 140.439, *p* < 0.0001; Tukey's test between control and oryzalin samples, *p* < 0.01; figure 11*n*). Moreover, the number of PVCs larger in area than 1 µm^2^ also increased considerably (up to 12%) with respect to control (2%) (figure 11*o*) and PVCs reached 5 µm^2^ in the presence of oryzalin (figure 11*o*). These larger PVCs showed the surface labelling observed also after Wm treatment (figure 11*j–m*), suggesting that they originate by homotypic fusion. After oryzalin treatment, YFP-SYP21 was occasionally mislocalized (about 10% of cells) to the apical PM, to vesicles clustered in the V-shaped inverted cone region (figure 11*g–i*, circle) and to the tubular compartments (figure 11*g–i*, arrows), suggesting substantial changes in membrane trafficking in the apex.

This increase in PVC dimensions and the concomitant decrease in YFP-SYP21 on tubular compartments after oryzalin treatment suggested that MTs could play a role in membrane trafficking between PVCs and tubular vacuoles and in promoting the fusion of PVCs with vacuoles.

In contrast, a low concentration of nocodazole, which affects short apical MT strands (electronic supplementary material, figure S7E), did not induce changes in pollen tube growth rate (electronic supplementary material, figure S2C) and in YFP-SYP21 distribution or PVC mean dimension (0.2 µm^2^) with respect to untreated pollen tubes (compare figure 8*a–c,g–i* with electronic supplementary material, figure S10A–H). In parallel, a significant increase in PVC mean area was observed in 0.05% DMSO-treated control cells with respect to untreated pollen tubes (ANOVA test: *F*_6.11498_ = 140.439, *p* *<* 0.0001, Tukey's test, *p* < 0.01; electronic supplementary material, figure S10G,H). Nocodazole treatment therefore reversed the effect of 0.05% DMSO on PVC dimensions in the apical and distal regions of the tubes (Tukey's test between control and nocodazole samples, *p* > 0.05), suggesting that this drug affects membrane trafficking in a different way from oryzalin.

In order to assess the effect of MT stabilization on PVC-vacuole trafficking as well, pollen tubes were incubated with 5 µM taxol. This treatment induced a weak decrease in pollen tube growth rate (Tukey's test, *p* > 0.05 with respect to SYP21-transformed and 0.85% DMSO-treated pollen tubes; electronic supplementary material, figure S2C). Microtubules in taxol-treated cells appeared as long bundles extending as far as the apex (electronic supplementary material, figure S7F). YFP-SYP21 labelling of tubular vacuoles decreased (electronic supplementary material, figure S10I–N) and PVC size increased with respect to untreated pollen tubes (electronic supplementary material, figure S10O–P; ANOVA test: *F*_6.11498_ = 140.439, *p* *<* 0.0001, Tukey's test between control and taxol samples, *p* < 0.01). A similar effect was observed in 0.85% DMSO-control cells (electronic supplementary material, figure S10O,P; Tukey's test between control and DMSO samples, *p* < 0.01), which showed more MTs in the cortical regions of the shank (electronic supplementary material, figure S7C). Taken together these results are strong evidence of a role of MTs in trafficking PVCs to tubular vacuoles.

To better define the role of MTs in trafficking towards vacuoles, colocalization experiments using YFP-SYP21 and CMAC were performed in control cells and in the presence of MT-active drugs (figure 10). After oryzalin treatment, the colocalization coefficient decreased significantly with respect to control (figure 10*a*; Student's *t*-test, *p* < 0.001 for apex/shank and for distal area). Moreover, colocalization points concentrated in PVCs, where they decorated the organelle-delimiting membrane (figure 9*f,p*; see arrowheads), and almost disappeared on tubular vacuoles, mainly in the distal region (figure 9*k–r*). Similarly, after oryzalin treatment the number of PVCs showing colocalization of SYP21 and CMAC increased significantly in the distal area (figure 10*b*; Student's *t*-test, *p* < 0.05; figure 9; arrows). Colocalization assays performed in the areas of pollen tubes with large vacuoles did not show any overlapping between CMAC and SYP21 in oryzalin treated pollen tubes (figure 9*i,f*, figure S8).

These findings suggest that MT depolymerization affects the delivery/fusion of PVCs to/with tubular vacuoles. Differences in PVC trafficking to vacuoles observed between the apex and distal area of the pollen tube could be due to different mechanisms regulating PVC/vacuole trafficking in these areas.

**Figure 12.**
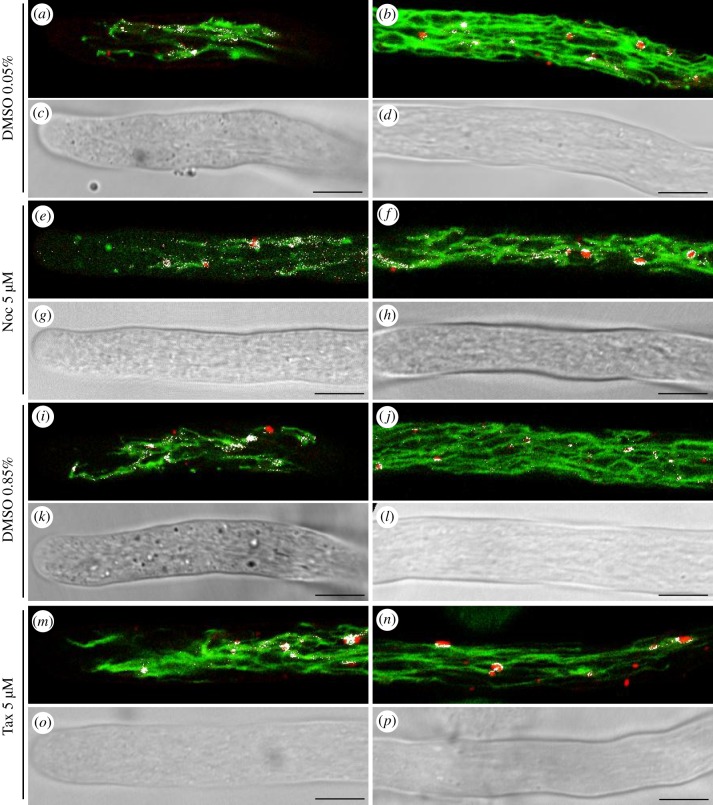
Colocalization of YFP-SYP21 and CMAC in pollen tubes treated with DMSO, nocodazole and taxol. (*a–d*) Colocalization of YFP-SYP21 and CMAC in the apex/shank (*a,c*) and distal area (*b,d*) of 0.05% DMSO-treated pollen tubes. Colocalized points are shown as white spots. (*e–h*) Colocalization of YFP-SYP21 and CMAC in the apex/shank (*e,g*) and distal area (*f,h*) of nocodazole-treated pollen tubes. (*i–l*) Colocalization of YFP-SYP21 and CMAC in the apex/shank (*i,k*) and distal area (*j,l*) of 0.85% DMSO-treated pollen tubes. (*m–p*) Colocalization of YFP-SYP21 and CMAC in the apex/shank (*m,o*) and distal area (*n,p*) of taxol-treated pollen tubes. All pictures show growing pollen tubes. Scale bar: 10 µm.

In pollen tubes treated with nocodazole, taxol or DMSO, the Pearson's coefficient did not vary significantly compared with control (Student's *t*-test, *p* > 0.05; figure 10*c,e*). However, in pollen tubes treated with MT-stabilizing agents (5 µM taxol or 0.05% and 0.85% DMSO), the colocalization points concentrated on PVCs and were missing on tubular vacuoles, especially in the distal area (figure 12*a–d*; *i–p*), suggesting a delay in fusion of PVCs with vacuoles. In addition, unlike with oryzalin, MT stabilization did not induce a significant increment in the number of SYP21-positive PVCs colocalized with CMAC (Student's *t*-test, *p* > 0.05; figure 10*f*), supporting the idea that the dynamics of MTs affects fusion of PVCs with tubular vacuoles.

Otherwise, nocodazole treatment reversed the effect of 0.05% DMSO and the distribution of colocalization points was similar to that on PVCs and tubular vacuoles of untreated pollen tubes (figure 12*a–d*; *e–h*). Interestingly, the number of SYP21-positive PVCs colocalizing with CMAC increased significantly in the apex of nocodazole-treated pollen tubes with respect to controls (figure 10*d*; Student's *t*-test, *p* < 0.001 versus control and 0.05% DMSO), while in the distal area this difference was not significant (figure 10*d*; Student's *t*-test, *p* > 0.05 versus control and 0.05% DMSO).

These findings confirmed that trafficking of PVCs to vacuoles was regulated differently in the distal and apical areas and that vacuole function could vary in different pollen tube regions. In addition, MTs could play different roles in the interaction of these organelles in the apical and distal areas of tobacco pollen tubes.

## 3. Discussion

This is the first report of a relationship between PVCs and tubular vacuoles in growing pollen tubes of *Nicotiana tabacum,* determined by means of the marker SYP21*.* Our results also highlight differences in the nature and function of tubular and large vacuoles during pollen tube growth. The use of drugs affecting the polymerization and dynamics of MTs also showed that PVC delivery to/fusion with tubular vacuoles are regulated differently by MTs in the apex/shank and distal areas. Besides these new insights into membrane trafficking and SYP21 function, the present findings also sustain, in addition to the prevalence of AFs for long-range movements, the emerging role of MTs in fine membrane positioning and organelle interactions in pollen tubes.

### 3.1. SYP21 localizes on PVCs and tubular vacuoles in tobacco pollen tubes

Two degradation pathways identified in tobacco pollen tubes appeared to be differentially regulated by AFs and MTs [6]. In particular, perturbation of MTs delayed the delivery of tip-internalized endocytic vesicles to vacuoles and mislocalized internalized cargoes to the Golgi apparatus [7].

To better clarify the relationship between MTs and the compartments involved in the degradation pathways, SYP21 was used as marker for PVCs [25,35]. SYP21 is a t-SNARE that has been localized on the PVC delimiting membrane in tobacco leaf protoplasts [24]. This is the first time that localization of SYP21 in tobacco pollen tubes has been reported. In transiently transformed cells, SYP21 localized on round organelles widely distributed in the cytoplasm but excluded from the clear zone. Control Wm, SYP21 overexpression and BP80 transformation experiments showed that SYP21-positive round compartments observed in tobacco pollen tubes are actually PVCs [24,36,37]. In line with observations in plant somatic cells [24,38], analysis of size frequencies showed that PVCs measured 0.05 to 0.7 µm^2^ in area. In addition, TEM observations and experiments of subcellular fractionation of pollen tubes revealed that membrane compartments recognized by SYP21 antibodies peaked in several fractions, confirming the presence of different populations of PVCs. Several populations of PVCs have also been reported in somatic cells [27,39].

Detailed analysis of the SYP21-positive PVC areas failed to detect any SYP21-PVCs in the 0.7–1.0 µm^2^ range in tobacco pollen tubes, suggesting that SYP21-positive PVCs increase in size by homotypic fusion, or by fusion of endocytic vesicles up to a maximum area of 0.7 µm^2^. The same size was observed in BP80-positive PVCs. Although PVCs with different dimensions could originate directly from the TGN, time-lapse imaging and multitracking analysis in *Arabidopsis* root hairs showed that PVCs interact together, tethering transiently in a movement defined as dancing-endosome interaction, which allows PVCs to come close together, fuse and separate, or fuse together in a stable manner, becoming larger [39]. The localization and dynamics of these PVCs were different in the apex and distal area of root hairs and were correlated with apical growth and endosome maturation. Late endosome fusion has also been observed in animals and yeasts [40–42], where spatio-temporal control of the process is postulated to prevent excessive enlargement of these compartments before fusion with lysosomes/vacuoles [41,42]. The regulation of endosome dimension, preventing excessive late endosome enlargement, ensures correct fusion with lysosomes [42]. In the pollen tube, analysis of the areas of SYP21 and BP80-positive PVCs suggested that a similar control mechanism could be present in tobacco pollen tubes.

Intriguingly, unlike GFP-BP80, SYP21 also localized on a network of highly dynamic tubules that extended longitudinally along the pollen tube as far as the apical area, without entering the clear zone. The morphology of this SYP21-tubular compartment resembled a tubular vacuole detected in *Arabidopsis* somatic cells by δ-TIP:GFP [43] and in tobacco and lily pollen tubes by the vacuolar marker CDFDA [7,31]. Double staining CMAC/YFP-SYP21 showed that SYP21 colocalized with CMAC in tubular vacuoles but never in large distal vacuoles, thus confirming the presence of SYP21 on tubular vacuoles and suggesting, for the first time, that tubular and large vacuoles are functionally different compartments.

The presence of SYP21 in vacuolar compartments was also supported by SYP21- and V-H^+^ATPase-positive organelles found in the high-density sucrose fractions of microsome sub-fractionation experiments. The localization of SYP21 on vacuoles is controversial since SYP21 was reported to be specific for PVCs in somatic cells [26,27], whereas more recently this protein was detected on PVCs and vacuoles [25,38,44]. Intriguingly, in the tobacco pollen tubes, 0.5 µM Wm induced homotypic fusion of tubular vacuoles and increased SYP21 fluorescence in these compartments. On the other hand, Wm did not induce heterotypic fusion between round PVCs and tubular vacuoles. This data supports the idea that tubular vacuoles could retain some of the functions of PVCs, although these compartments appeared as different organelles.

In plants, vacuole fusion induced by Wm has only been observed in the *vti11* mutant (impaired tonoplast trafficking mutant - itt3/vti11) [45]. VTI11 is a SNARE complex polypeptide involved in homotypic vacuole fusion during vacuole biogenesis [37,45], which in plant somatic cells requires the fusion of tubular provacuoles derived from ER [46–48]. Analogously, the fusion of tubular vacuoles induced by Wm in tobacco pollen tubes suggests that this compartment could have provacuole-like identity. As a matter of fact, CMAC staining showed that in the more distal region of pollen tubes, tubules fused together and participated in large vacuole biogenesis. SYP21 did not colocalize with CMAC in these large vacuoles, suggesting that the delimiting membrane protein composition of tubular vacuoles changes before they coalesce into the large vacuole. It is also possible to imagine that SYP21 and other proteins could be recycled from the tonoplast back to PVCs or to the TGN to be reused in post-Golgi secretion.

Similar tubular vacuoles have also been observed in filamentous fungi having polarized growth [49]. In basidiomycetes and mycorrhizal fungi, tubular vacuoles play a role in bidirectional solute transport along hyphae, on a scale in the millimetre to centimetre range [50–52]. It cannot be excluded that this organelle functions in the long-distance transport of molecules in fungi and pollen tubes alike. In addition, the tubular vacuole in fungi is proposed to function as an endosomal compartment. In fact, t-SNARE AoVam3p localizes on the delimiting membrane of these tubular vacuoles as well as in late endosomes/PVCs. AoVam3p is the homologue of PEP12 from *Aspergillus oryzae* [53] and PEP12/SYP21 in plants [26]. The similar localization of AoVam3p and SYP21 suggests that tubular vacuoles could also function as endosomal compartments in pollen tubes.

Previous data on SYP21 function was controversial: although it was reported that *syp21* mutant cannot be rescued by expression of SYP22 in *Arabidopsis* somatic cells [35,54], other studies showed that SYP21 has interchangeable functions with SYP22 and SYP23 in PVC-vacuole fusion [25,38]. The presence of SYP21 on tubular vacuoles suggests that this SNARE could have a specific role in heterotypic fusion of PVCs with vacuoles or in mediating trafficking from the ER/TGN during vacuole biogenesis. Different pathways for protein delivery to vacuoles were recently identified in somatic cells, and involved direct trafficking from the ER or AP3-dependent/PVC-independent pathway [55–57]. However, these alternative pathways are still uncharacterized in pollen tubes.

### 3.2. MTs interact with organelles involved in degradation pathways

It is known that AFs mediate long transportation movements in somatic plant cells, while MTs are involved in the fine positioning of membrane compartments [14,15]. In pollen tubes, AFs and MTs both contribute to the transport of endosomes. Previous studies on endocytosis revealed that AFs are involved in shank-internalized PM trafficking, while MTs play a role in endocytosis, membrane sorting in the tip and transport of tip-internalized endocytic vesicles towards the vacuole [7].

To better characterize the role of MTs in the degradation pathways of tobacco pollen tubes, we used different *in vitro* and *in vivo* approaches that revealed direct interaction of organelles with purified MTs and differences in PVC behaviour under MT-perturbing drugs. The direct interaction between MTs and PVC *in vitro* was specific and ATP-dependent.

In pollen tubes, it has been shown that interaction of MTs with organelles could be mediated by motor proteins such as kinesins [58–61]. Kinesins stably bind to MTs in the presence of the ATP non-hydrolysable analogue AMP-PNP, and are released by ATP [62]. In fact, AMP-PNP has been widely used to purify MT motor proteins from a wide range of organisms [62,63]. The binding of SYP21-positive organelles to MTs was also AMP-PNP-enhanced and ATP-inhibited, suggesting that binding of SYP21-positive organelles was probably mediated by an ATP-dependent motor protein. In addition, TEM observations of MT-bound organelles suggested that this interaction could be mediated by particles decorating the membrane surface. These particles could be protein complexes that allow organelle–MT interaction. In somatic cells, CLASP has also been shown to be a plus-end-tracking MT-associated protein connecting sorting endosomes to MTs, probably by interacting with SNX1 [64]. This mechanism may also participate in the interaction of membrane compartments with MT extremities. Further experiments to characterize SYP21-positive endosome proteins, responsible for the interaction with MTs, are therefore needed to clarify this point.

Membrane fractionation experiments in the presence of oryzalin showed different migration of PVC compartments in the sucrose density gradient, supporting the idea that disturbance of MTs modifies PVC trafficking. In addition, weak modification in the distribution of Bip1 and Arf1, markers for ER and Golgi apparatus, respectively, also suggests a possible interaction between these compartments and MTs. In fact, MTs are reported to play a role in the maintenance of Golgi and ER morphology [14,16,18,65].

### 3.3. MTs play a role in PVC delivery to and/or fusion with tubular vacuoles

In transiently transformed pollen tubes, MT depolymerization by oryzalin caused SYP21 to accumulate on PVCs and almost disappear on tubular vacuoles. This effect was accompanied by PVC enlargement. It could be hypothesized that MTs affect the dynamic of dancing-endosome interactions [39] causing PVCs to fuse together in a stable manner so inducing the PVC enlargement. This could be an important cue to clarify the dynamic of these PVC interactions.

The PVC enlargement and the disappearance of SYP21 on tubular vacuoles together with colocalization analysis suggested that MT depolymerization affects PVC delivery to and/or fusion with tubular vacuoles. To be sure that PVC modification was due to MT depolymerization, preliminary experiments to check the integrity of AFs were performed following oryzalin treatment. Transient transformation using pLAT52/Lifeact-mEGFP did not show any detectable modification of AF pattern in the presence of oryzalin with respect to the control. On the other hand, since it is known that AFs play a major role in membrane movements in pollen tubes, it could also be worthwhile to do trafficking experiments in the presence of latrunculin B (LatB). However, in *Nicotiana tabacum* pollen tubes, 5 nM LatB dramatically altered MT pattern in the shank and tip. The AF-perturbing drug would therefore not provide unambiguous results on the actual role of AFs in PVC trafficking. As AFs and MTs are known to be intimately linked [66], we obviously cannot exclude cooperation between AFs and MTs in this process. Further experiments using different approaches could help to clarify this point.

Like MT depolymerization, MT stabilization by taxol induced similar changes in the localization of SYP21 on PVCs and tubular vacuoles. However, unlike after oryzalin treatment, Pearson's coefficients were similar in controls (untreated and 0.85% DMSO-treated) and taxol-treated cells, suggesting that MT stabilization may affect trafficking of SYP21 to PVCs, as well as PVC delivery to and/or fusion with vacuoles. In contrast, the number of PVCs that colocalized with CMAC was not enhanced in taxol-treated pollen tubes, even if their fusion with vacuoles was impaired. This supports the idea that MT depolymerization, but not MT stabilization, plays a role in the proper positioning of PVCs near the tubular vacuole to induce PVC/vacuole fusion.

Area frequencies and the results of fractionation experiments suggested that PVCs undergo modification before fusing with vacuoles and that MTs could be involved in this event. In *Arabidopsis* stem cells, PVC maturation was impaired by oryzalin and by FAB1/PIKfyve inhibitor, suggesting that endosome maturation requires recruitment of effector molecules, such as Ara7 and SNX1, by PI(3,5)P_2_, as well as interaction of endosomes with cortical MTs [67,68]. Also in tobacco pollen tubes, perturbation of MTs may possibly affect the control of PVC size and PVC membrane/content composition.

The emerging idea is that MTs play a major role in different processes in pollen tube degradation pathways: MTs could primarily favour the delivery and/or fusion of PVCs with tubular vacuoles. As stated for trafficking of cellulose synthase complex (CSC), where cortical MTs play a role in positioning and targeting Golgi-derived small CSC vesicles for correct fusion with the PM [14,15,69], MTs could play a similar role in properly localizing PVCs near vacuoles to promote fusion events. The presence of colocalized points between YFP-SYP21 and CMAC on the surface of PVCs leads to the hypothesis that these compartments are transported near tubular vacuoles to promote fusion. The increase of colocalized PVCs after oryzalin and nocodazole, with respect to taxol, strongly supports that the MT dynamics is responsible for the proper positioning of PVCs with respect to tubular vacuoles and for the appropriate timing to allow fusion events.

In the emerging model, SYP21-PVCs move in the cytoplasm thanks to the AF dependent cytoplasmic streaming and are captured by MTs whose plus end dynamic instability could be responsible for positioning PVCs near tubular vacuoles to promote their fusion. MT perturbing agents prevent the proper positioning of PVCs and thus the fusion events between PVCs and tubular vacuoles. The use of a MT-plus-end marker could better clarify this process.

In addition, MTs could also be involved in PVC modification by recruitment of effectors leading to PVC membrane and content changes. However, this hypothesis should be further investigated.

Experimental evidence also suggests that MTs influence PVC trafficking differentially in the apex/shank and distal regions. The different colocalization of SYP21-CMAC in tubular vacuoles of the tip/shank and distal area after MT drug treatments suggests that tubular vacuoles may have different functional domains along the pollen tube. In the pollen tube apex/shank, the delivery of PVCs to vacuoles may possibly not be inhibited completely by depolymerization of MTs. Alternatively, SYP21 could also reach the tubular vacuoles by MT-independent pathways. In fact, the journey of post-Golgi-secreted proteins involved in vacuole biogenesis and that of endocytic vesicles towards the vacuole may not involve PVCs or the TGN [5,55,56].

Differences between distal and apical PVC–tubular vacuole interaction were also highlighted by nocodazole treatment. Unlike oryzalin, which completely depolymerizes MTs along the tube, nocodazole increases the rate of GTP hydrolysis in the E-site of the β-tubulin subunit [70], thus increasing the time that MTs spend in pause and catastrophe phases in the apex/shank, leaving MTs in the distal areas unaltered [71].

While oryzalin increased the number of CMAC-stained SYP21-positive PVCs in the distal area, nocodazole was associated with a significant increase in the number of PVCs colocalizing with CMAC in the apex/shank, without any difference with respect to control in the distal area. These findings suggest that different MT populations affect PVC trafficking differently. Moreover, while DMSO appeared to improve homotypic PVC fusion and impair PVC delivery to and/or fusion with vacuoles in the apex/shank and distal regions, nocodazole reversed this effect, apparently inducing an increase in PVC–vacuole fusion events compared with DMSO, and preventing PVC enlargement. The increasing trend of the SYP21-CMAC colocalization coefficient in nocodazole-treated pollen tubes, though not significant, also suggests the existence of different pathways for SYP21 trafficking/recycling which were enhanced by nocodazole. Altogether, these observations suggest that dynamic MTs play a role in controlling PVC trafficking and delivery to proper position near the tubular vacuoles in the apex/shank. In the distal area, where long MT bundles were apparently not disturbed by nocodazole, PVC delivery to vacuoles was less affected. It could be interesting to investigate whether the behaviours of MTs in PVC trafficking in the apex/shank and distal area are influenced by different relationships between MTs and AFs.

The different trafficking observed for SYP21 and PVCs in the apex/shank and distal area of pollen tubes could reflect different functional significances of the degradation pathways in these regions. Prevacuolar compartments and tubular vacuoles in the apex/shank could possibly be involved mostly in endocytosis, and PVCs could play a major role in fast delivery of material destined for degradation to tubular vacuoles. Otherwise, PVCs and tubular vacuoles in the distal area could both participate in endocytosis and in biogenesis of large vacuoles. SYP21 could be retrieved from tubular vacuoles to PVCs/TGN before they coalesce into large vacuoles in the distal regions of the tubes.

## 4. Conclusion

The model emerging from these results predicts the involvement of MTs in trafficking to tubular vacuoles in tobacco pollen tubes. The use of SYP21 allowed us to identify, for the first time, PVCs and tubular vacuoles as crucial steps in the MT-dependent degradation pathway. Studies using different drugs affecting the polymerization status of MTs showed that different MT populations play specific roles in PVC trafficking and in PVC delivery to and/or fusion with tubular vacuoles in the apex/shank with respect to the distal regions. Furthermore, tubular vacuoles emerge as a multifunctional compartment, being involved in endosomal trafficking and in the biogenesis of large vacuoles.

## 5. Material and methods

### 5.1. Fluorescent probes and drugs

Wortmannin (Sigma) was dissolved in DMSO to a concentration of 10 mM and then diluted to 0.5 µM final concentration in the culture medium. Nocodazole (methyl 5-(2-thienylcarbonyl)-1 h benzimidazol-2-yl) (Fluka, USA) stock solution in DMSO had a concentration of 10 mM and was used in culture medium at a final dilution of 5 µM. Taxol (Paclitaxel; Sigma) was dissolved in DMSO to a concentration of 585 µM and then diluted to 5 µM final concentration in the culture medium. Oryzalin (methyl 5-(2-thienylcarbonyl)-1 h benzimidazol-2-yl) stock solution in water had a concentration of 1.38 M and was used in culture medium at a final dilution of 0.1 µM. Blue-CMAC (7-amino-4-chloromethylcoumarin) was resuspended in DMSO to a concentration of 10 mM and then diluted to 2 µM final concentration in culture medium.

### 5.2. Pollen tube growth

*Nicotiana tabacum* (L.) pollen was collected from plants in the Botanical Garden (Città Studi) of Milan University in summer and stored at –20°C. For biochemical, TEM and immunolabelling investigations, pollen grains were cultured in BK medium [72] supplemented with 12% (w/v) sucrose at 23 ± 2°C as reported by Moscatelli *et al*. [6]. For transient gene expression, pollen grains were collected from fresh flowers of *N. tabacum* (L.) and allowed to germinate at 23°C on solid medium as reported by Kost *et al*. [73].

### 5.3. Tubulin purification and preparation of taxol-stabilized microtubules

Microtubules from bovine brain were obtained by three cycles of temperature-dependent polymerization and depolymerization as reported by Williams and Lee [74]. Tubulin was purified by HiTrap Q XL anion exchange column (GE Healthcare, Uppsala, Sweden). Proteins were eluted with a KCl gradient from 0 to 1 M and tubulin was eluted at 0.55 M KCl. The fractions from 0.3 M KCl and 1 M KCl were analysed by SDS-PAGE and those with higher tubulin concentration were pooled. After addition of 1 mM GTP, the tubulin pool was assayed (Bradford method) for protein concentration using BSA as standard and frozen in liquid nitrogen before storage at −80°C. For binding experiments, MTs were polymerized from monomeric tubulin (10 mg ml^−1^) in the presence of 1 mM GTP, 10% glycerol and 30 µM taxol at room temperature for 30 min. The microtubule sample was centrifuged at 12 000*g* for 50 min at 20°C and resuspended in Tx-PEM buffer (80 mM Pipes, pH 6.8, 1 mM MgCl_2_, 1 mM EGTA and 5 µM taxol).

### 5.4. Microsome purification with and without peripheral protein stripping

Tobacco pollen tubes were grown in liquid BK medium for 2 h and then rinsed with 10 ml HEM buffer pH 7.4 (25 mM HEPES, 2 mM EGTA, 2 mM MgCl_2_, 0.5 mM EDTA, 1 mM dithiothreitol (DTT), 1 mM phenylmethylsulfonyl fluoride (PMSF), 10 µg ml^−1^ TAME, 10 µg ml^−1^ leupeptin, 10 µg ml^−1^ pepstatin A, 4 µM aprotinin, 8 µM antipain) containing 12% sucrose. After centrifuging at 2000 r.p.m. for 10 min at 10°C in a Beckmann JS13.1 rotor, pollen tubes were homogenized on ice in two volumes of HEM buffer containing 10% mannitol. The homogenate was centrifuged at 572*g* for 4 min at 4°C and the supernatant loaded onto a 0.5 M sucrose cushion (3 ml) in HEM buffer and centrifuged at 64 200*g* for 23 min at 4°C. The pellet containing microsomes (P2) was resuspended in PEM buffer. Aliquots of P2 and supernatant (S2) were protein assayed (Bradford method) using BSA as standard protein. For peripheral protein stripping, P2 was incubated with 0.8 M KCl in HEM buffer, loaded onto a 0.5 M sucrose cushion in HEM buffer and centrifuged at 64 200*g* for 23 min at 4°C. These organelles, resuspended in HEM buffer, were used for binding experiments.

### 5.5. Binding assay with AMP-PNP or ATP

MTs polymerized as described above (100 µg protein) were incubated with microsomes (40 µg protein) for 30 min at room temperature (+MT). The microsomes had previously been incubated for 15 min with buffer, 5 mM AMP-PNP or 1 mM ATP and 5 µM taxol. Stripped microsomes (see above) were incubated with AMP-PNP. +/−MT-microsome samples were then centrifuged for 1 h at 33 300 r.p.m. in a Beckman SW60 rotor, at 20°C through a 1.2 M sucrose cushion to separate MTs bound to organelles from those not bound to organelles. Three different fractions were collected: soluble fraction (S), organelles lying on the cushion (I) and pelleted MTs and organelles (P). In parallel, a control sample without MTs (−MT) was treated as described for MT/microsome incubation. The P fractions with and without MTs (P +MT and P –MT, respectively) were used for TEM analysis or denatured for electrophoresis. Binding experiments carried out using AMP-PNP were repeated 25 times. Seven and 10 independent experiments were performed by using ATP and stripped microsomes, respectively. About 100 TEM images were taken for each experiment.

### 5.6. Sucrose density gradients

Pollen tubes grown on liquid BK medium were incubated with 0.1 µM oryzalin for 15 min. Microsome fractions obtained by growing pollen tubes in the absence (control) or presence of oryzalin were centrifuged through a continuous 15% to 65% sucrose gradient at 50 000*g* for 16 h. The sucrose gradient was obtained by three freezing/defrosting cycles of 40% sucrose solution in 50 mM imidazole, pH 7.5, 2 mM EDTA, 1 mM PMSF and 1 mM DTT. After centrifugation, fractions of 0.5 ml were recovered from the top of the gradient and then stored at −80°C or denatured for electrophoresis. Protein concentrations were assayed (Bradford method) using BSA as standard protein. Fractionation experiments were repeated three times.

### 5.7. SDS-PAGE and western blot analysis

SDS-PAGE analysis was performed using 10% linear acrylamide concentration according to the method of Laemmli [75]. Gels were stained with Coomassie Brilliant Blue R250. Western blot was performed according to Towbin *et al*. [76]. Arf1, V-H^+^ATPase, H^+^ATPase (Agrisera, Sweden), GRP78/Bip (Sigma) and SYP21 antibodies (kindly provided by N. Raikhel, University of California Riverside, USA) were used at final dilution of 1 : 2000 and detected as outlined in the Amersham ECL kit booklet. All gels and western blot images were scanned using Epson Perfection V750 PRO and Adobe Photoshop software. Quantification of protein levels was carried out with Quantity One Software, using the Volume tools to quantify bands, and the results were calculated as Adjusted Volume (volume minus background) of immunoreactive bands normalized to values obtained for tubulin in the same immunoblots.

### 5.8. pLAT52:YFP-SYP21, pLAT52:YFP:NOS and pLAT52-GFP:BP80 plasmid construction

The *LAT52* promoter-driven *YFP:SYP21* construct was generated by directional cloning. *YFP:SYP21* was removed from the *pC130035S:YFP:SYP21* construct [77] by *XbaI/SacI* digestion that was also used to remove *mGFP4:RabA4d:NOS* from the *pUC19LAT52:mGFP4:RabA4d:NOS* construct [78]. The *YFP:SYP21* region from *pC130035S:YFP:SYP21* was inserted into the cohesive *XbaI* and *Sac*I ends of the *pUC19LAT52* plasmid to create the translational fusion *LAT52*:*YFP:SYP21*. For the generation of the *pLAT52-GFP:BP80* construct, the Lat52 promoter (0.6 Kb) was moved as an XhoI-Bam HI fragment from pLat52-GFP-TUB6 [7] to pESC-LEU (Genbank accession #AF063849) for the sole purpose of providing a Hind III site next to XhoI. Next, the promoter was moved as a Hind III-BamHI fragment from pESC-LEU to pSGFP491 [8] using the same restriction sites to open the recipient plasmid, thereby substituting the 35S promoter with the Lat52 promoter. *NotI/SacI* digestion was used to remove the *SYP21* gene from the *LAT52* promoter-driven *YFP:SYP21:NOS* construct in order to create the *pUC19 LAT52*:*YFP:NOS* plasmid. Non-compatible sticky ends, produced by the double digestion *NotI/SacI*, were blunted using Klenow fragment. To separate the vector away from the *SYP21* gene cut out of it, we loaded the plasmid DNA on 0.8% agarose gel and purified the plasmid backbone bands using the QIAquick Gel Extraction Kit. The recovered plasmid backbone was used for self-ligation and transformation. The finished *pUC19 LAT52*:*YFP:NOS* construct was verified by sequencing and used for control experiments.

### 5.9. Transient gene expression and MT drug treatment

Expression vectors (1.2 µg pLAT52:YFP-SYP21, 1.5 µg pLAT52:YFP-SYP21 for overexpression, 1.5 µg pLAT52:GFP-BP80 and 0.78 µg pLAT52:Lifeact-mEGFP) were transferred to mature pollen grains on solid culture medium (see pollen tube growth) using a helium-driven particle accelerator (PDS-1000/He; Bio-Rad, Hercules, CA, USA). Pollen grains were placed under the stopping screen at a distance of 8 cm and bombarded in a 28″Hg vacuum using a helium pressure of 1100 psi, according to the manufacturer's recommendation (Bio-Rad) [79]. Gold particles (1 µm) were coated with plasmid DNA as described by Kost *et al*. [73]. Bombarded cells were kept at 23°C in the dark for 5 h before observation. Microtubule-active drugs were added to solid culture medium and the pollen tubes were incubated for 5 min with 0.1 µM oryzalin and for 15 min with 5 µM nocodazole, 5 µM taxol, 0.5 µM Wm and 0.05% or 0.85% DMSO. Twenty transformation experiments were performed using pLAT52:YFP-SYP21 without drug treatments, while for each treatment three to five different experiments were done. Three different experiments were performed for pLAT52 : GFP-BP80 transformation. For each transformation experiment at least 20 pollen tubes were analysed.

### 5.10. Colocalization experiments

Pollen tubes were transiently transformed with pLAT52:YFP-SYP21 and grew as described above approximately 6 h after bombardment. Blue-CMAC 2 µM (Molecular probes, Invitrogen) was added to transiently transform pollen tubes on solid culture medium for 15 min. The dye was then removed and the tubes were rinsed by adding liquid culture medium to the solid culture medium for 5 min. Microtubule-active drugs were added to solid culture medium as described for transient gene expression. The fluorescence observations were carried out using a Leica TCS SP2 microscope with a 63× oil immersion (NA 1.4) objective (Leica Microsystems, GmbH, Wetzlar, Germany). The UV and 488-nm laser lines were used to excite Blue-CMAC and YFP, respectively, and the fluorescence was collected in the 440–480 and 520–550 nm emission windows to acquire Blue-CMAC and YFP, respectively. Images were recorded in sequential scan mode of the LCS software (Leica Microsystems, GmbH, Wetzlar, Germany) and aligned by Leica LASx software. Colocalization analysis was carried out using the JACoP plug-in of ImageJ software [80]. The degree of colocalization was evaluated by calculating Pearson's coefficient [80]. For visualization purposes, pixels with intensities exceeding user-defined thresholds for both channels were represented as white spots in overlapped images (colocalized points). Pearson's coefficients were calculated for each experiment using the same ROI in all images. For colocalization experiments between YFP-SYP21 and GFP-BP80 we used 514 nm and 458 nm laser lines to excite YFP and GFP, respectively; the fluorescence was detected in the 560–610 nm and 480–520 nm emission windows respectively, following the procedure reported above. For YFP-SYP21/CMAC colocalization, three to five different experiments were performed for each drug treatment. For YFP-SYP21/GFP-BP80 cotransformation, three different experiments were done. For each transformation experiment at least 20 pollen tubes were analysed.

### 5.11. Microtubule labelling

Microtubules were detected in pollen tubes grown in BK medium as control or in BK medium spiked with nocodazole (5 µM), DMSO (0.05%, 0.85%), oryzalin (0.1 µM), taxol (5 µM), Wm (0.5 µM) and latrunculin B (5 nM). Samples were then incubated in fixing solution (3.7% formaldehyde, 10% sucrose, 100 mM PIPES, 5 mM MgSO_4_, 0.5 mM CaCl_2_, 0.01% MBS pH 7.0) with drugs to prevent MT recovery during the early stages of fixation. Indirect immunofluorescence was performed as described in Idilli *et al*. [7] using the anti-α tubulin monoclonal antibody TUB 2.1 (purchased from Sigma, USA) at a concentration of 1 : 200. An FITC-conjugated anti-mouse secondary antibody was used at 1 : 200 final concentration (Invitrogen, USA). Optical sections (0.5 µm) and three-dimensional projections of specimens were obtained with a Leica TCS NT confocal microscope with a 40× objective for imaging. All images were recorded using a stepper motor to make Z-series.

### 5.12. Transmission electron microscopy and immunogold labelling

TEM analysis was performed on P fractions obtained in binding experiments with or without microtubules (P +MT and P –MT, respectively). Control samples (with AMP-PNP) and KCl- or ATP-treated samples were fixed to formvar carbon-coated nickel grids for 30 min and then quickly rinsed once with TX-PEM. After 10 min fixation in 2% glutaraldehyde, the grids were rinsed three times with 5 mM EGTA and negative stained with 1% uranyl acetate for 10 s. Immunogold labelling was performed on pollen tubes grown for 60 min in BK medium and processed for fixation by the protocol reported in Moscatelli *et al*. [5]. Seventy-nanometre ultra-thin sections, obtained using a Reichert Jung Ultracut E microtome, were collected on formvar carbon-coated nickel grids. Sections were blocked by incubation with 1% BSA for 1 h and, after three rinses of 5 min each in TSB, SYP21 antibody was used at a final concentration of 1 : 1500 for 2 h at room temperature. Grids were rinsed once in TBS with 0.1% Tween20 and twice in TBS and then incubated with 10 nm gold-conjugated goat anti-rabbit IgG (BB International, USA) diluted 1 : 100 in TBS. After three rinses with TBS, grids were incubated with 1% glutaraldehyde for 10 min, rinsed with distilled water and stained with 3% (w/v) uranyl acetate for 30 min. Observations were performed with an EFTEM LEO 912AB transmission electron microscope (Zeiss, Jena, Germany) operating at 80 kV.

### 5.13. Statistical analysis

Differences in Syp 21-compartment areas between treatments were analysed by Student's *t*-test and analysis of variance (ANOVA). Tukey's *post hoc* test of honestly significant difference (HSD) was used to sort all differences between treatments. Frequency distributions of SYP21-compartment areas were calculated and represented by box plots and histograms. All analyses were performed with IBM-SPSS Statistics 22 (IBM SPSS Inc., Chicago, IL, USA). The letters a and b in the graphs indicate significantly different values.

## Supplementary Material

Supplementary figures

## Data accessibility

This article has no additional data.
