# Supplementary figures and images for: Microtubules play a role in trafficking prevacuolar compartments to vacuoles in tobacco pollen tubes
Source: Open Biol. 2018 Oct 31;8(10):180078. doi: 10.1098/rsob.180078 (PMC6223213; doi:10.1098/rsob.180078)

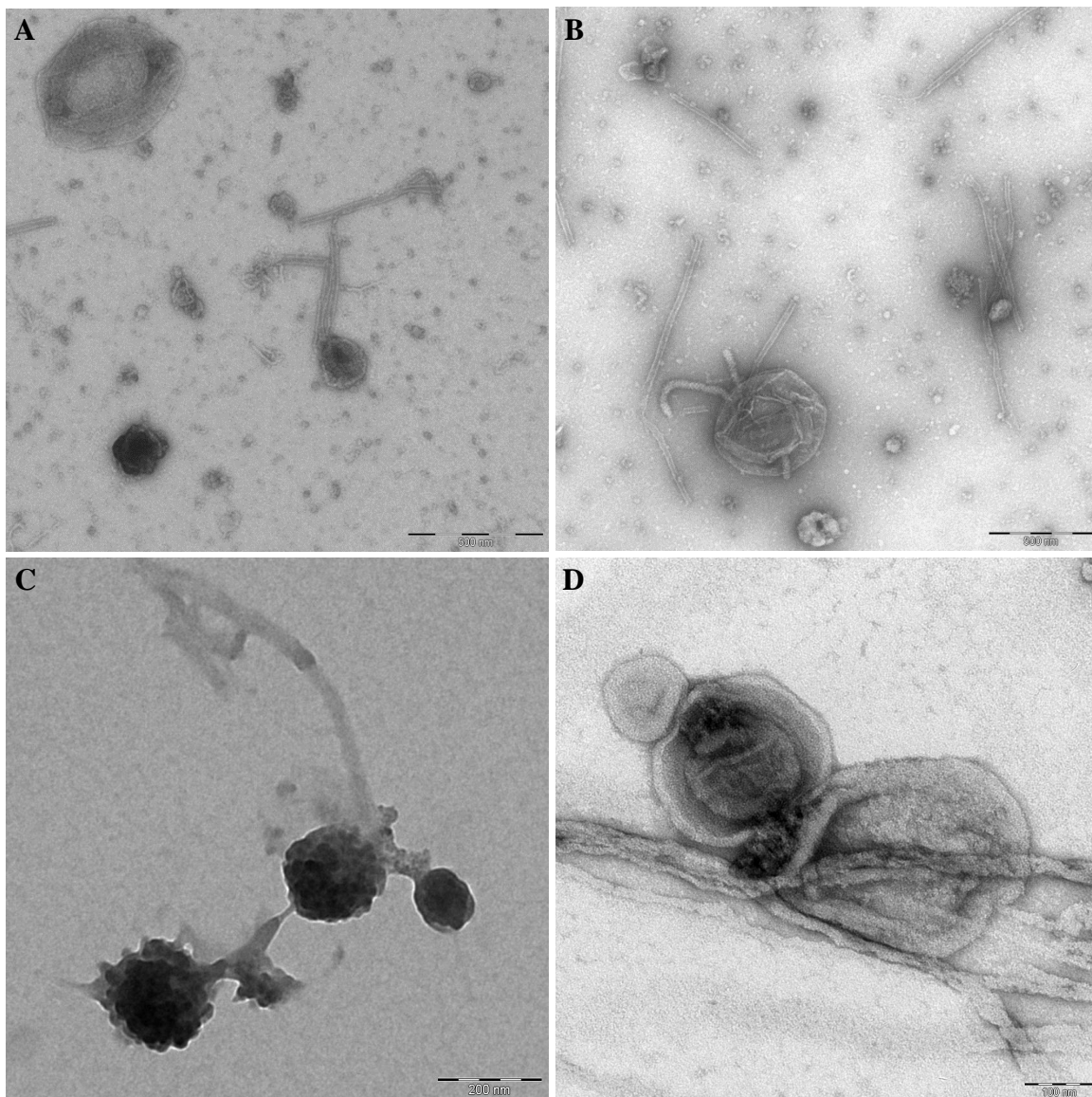

**Figure S1**

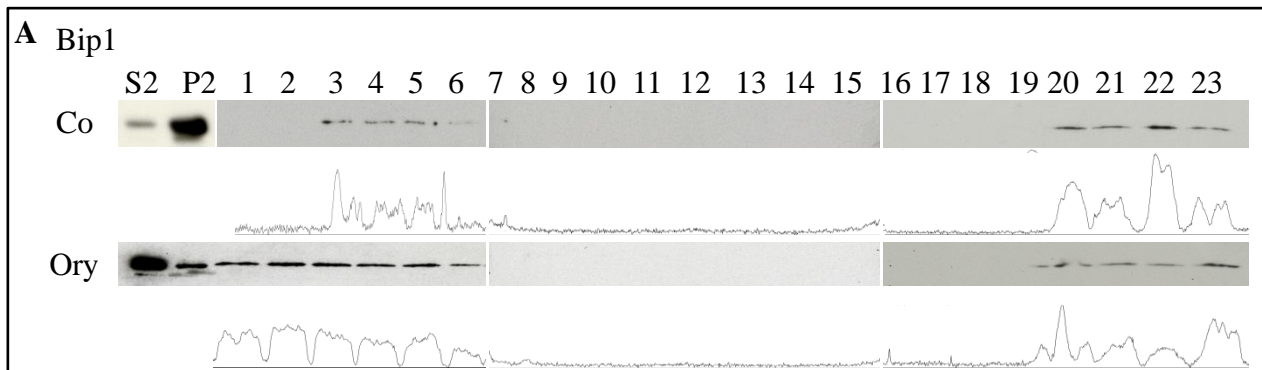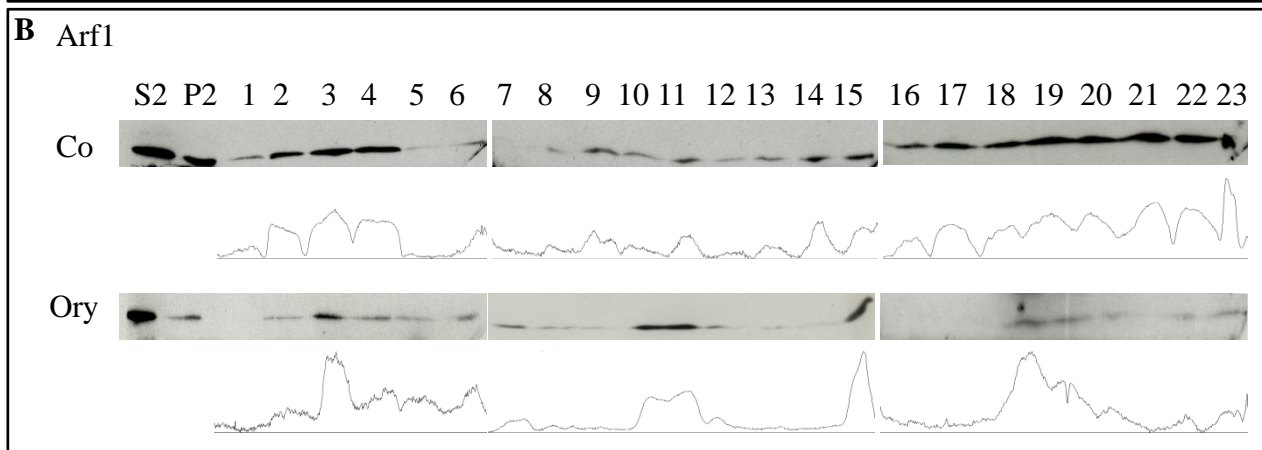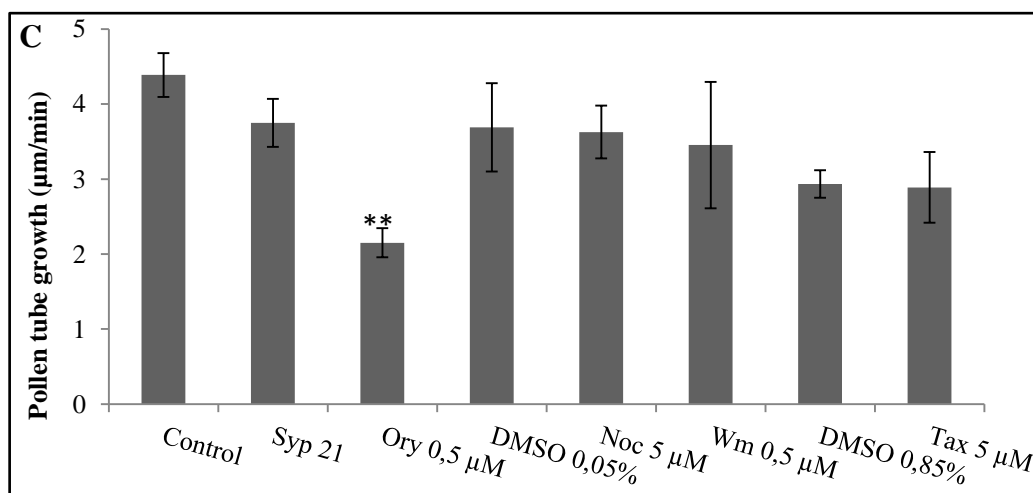

**Figure S2**

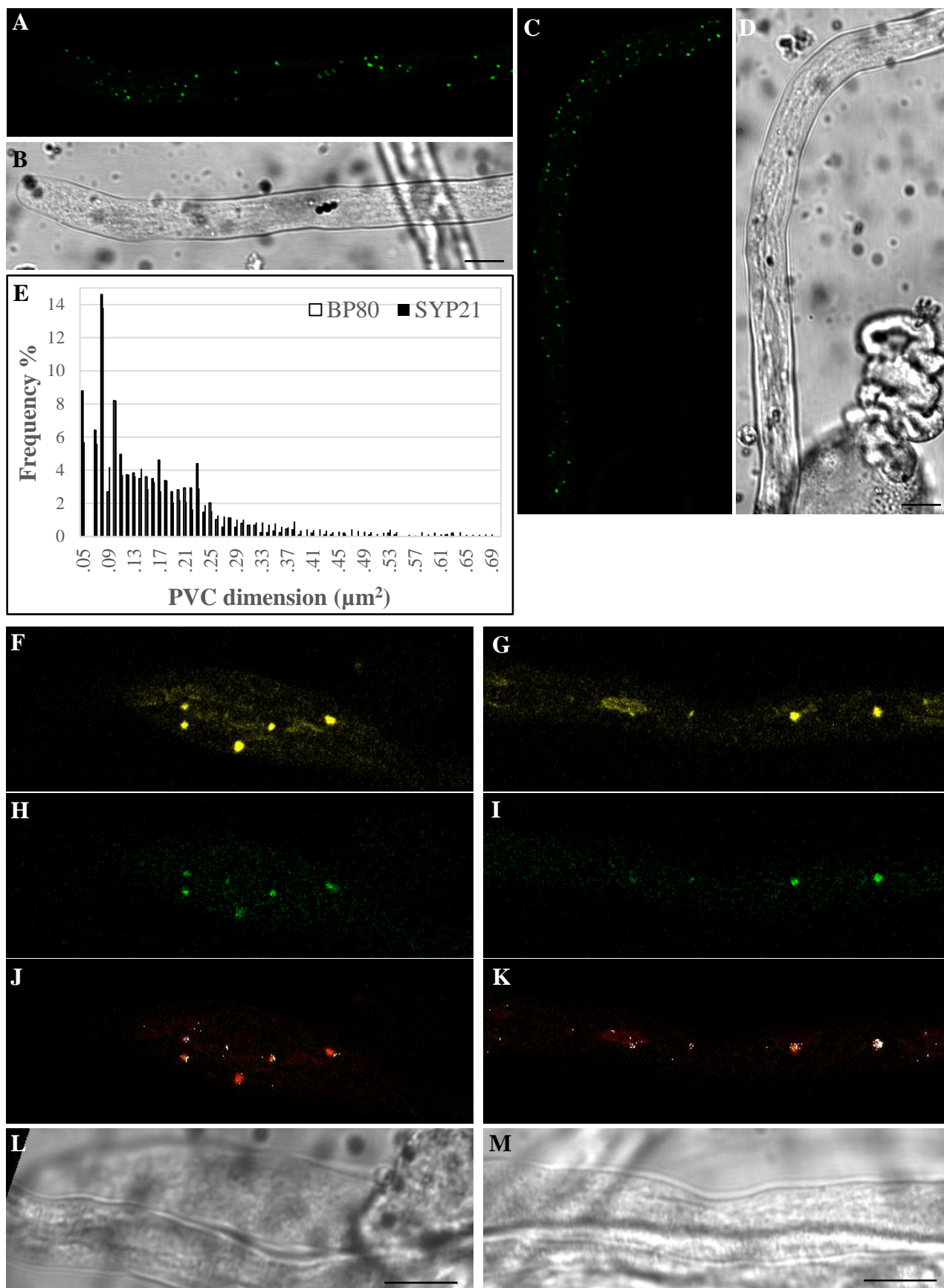

Figure S3

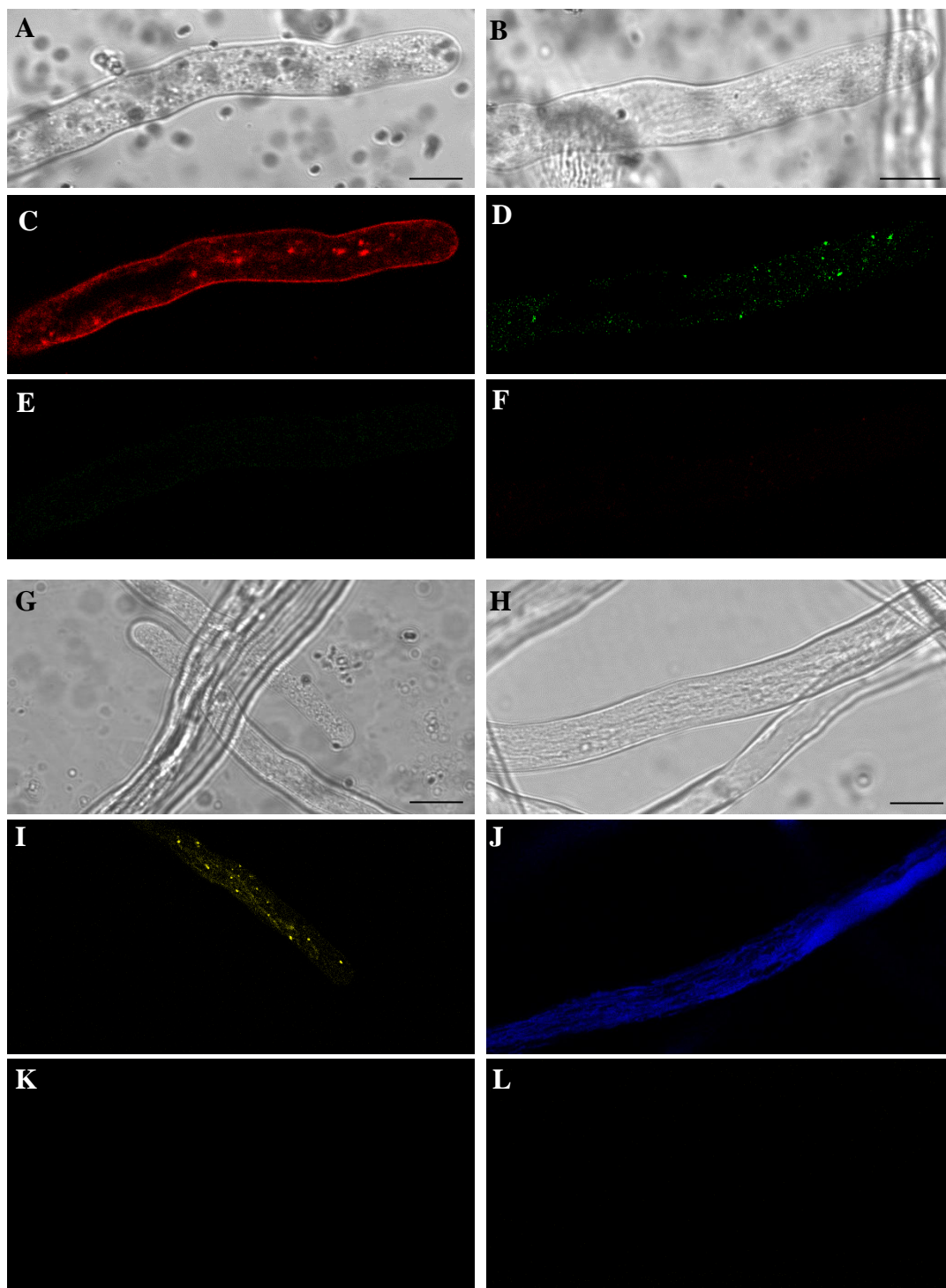

**Figure S4**

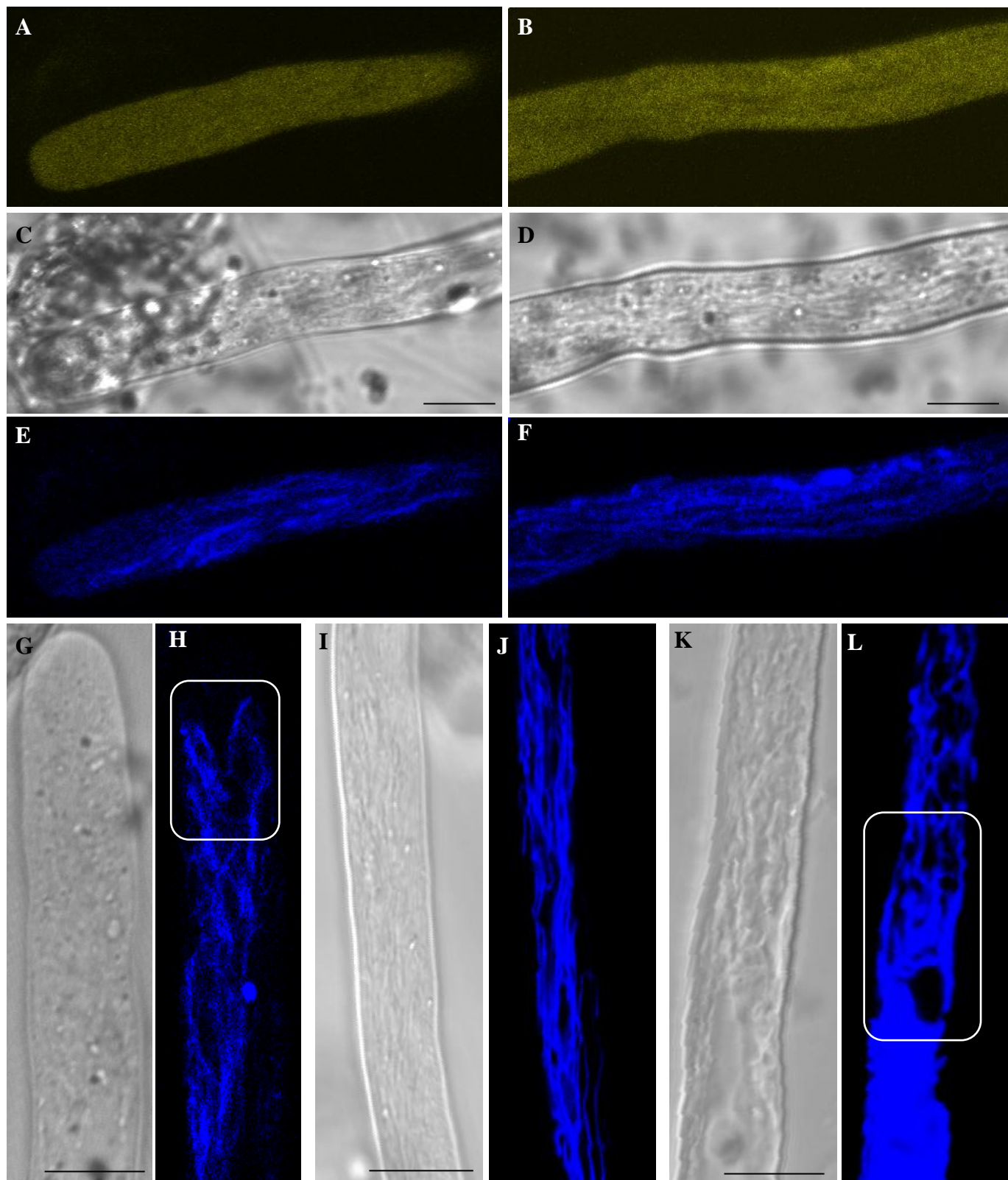

**Figure S5**

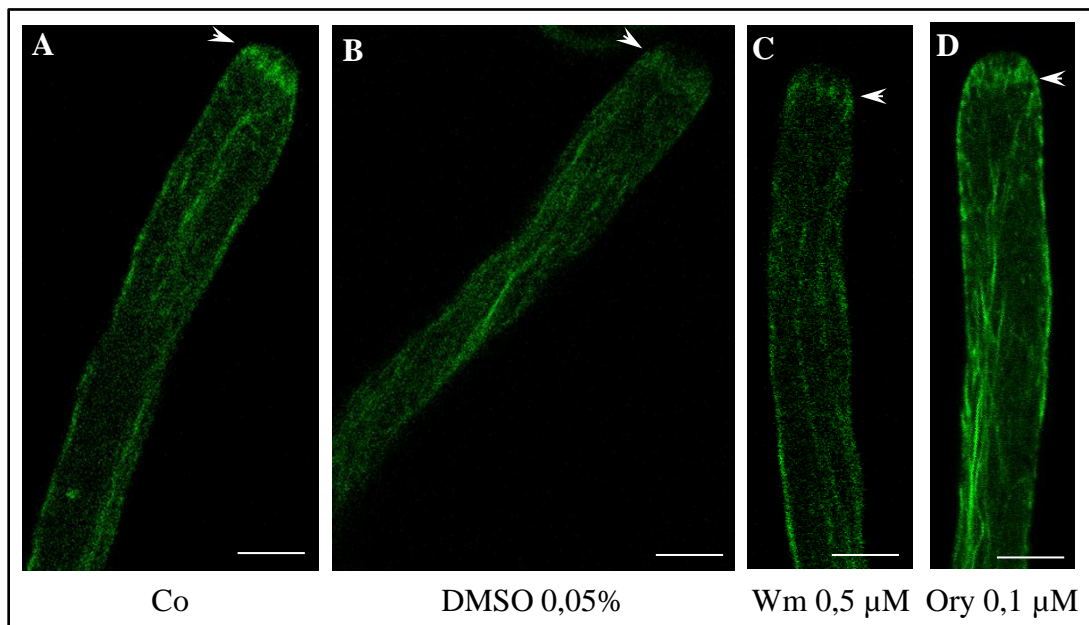

**Figure S6**

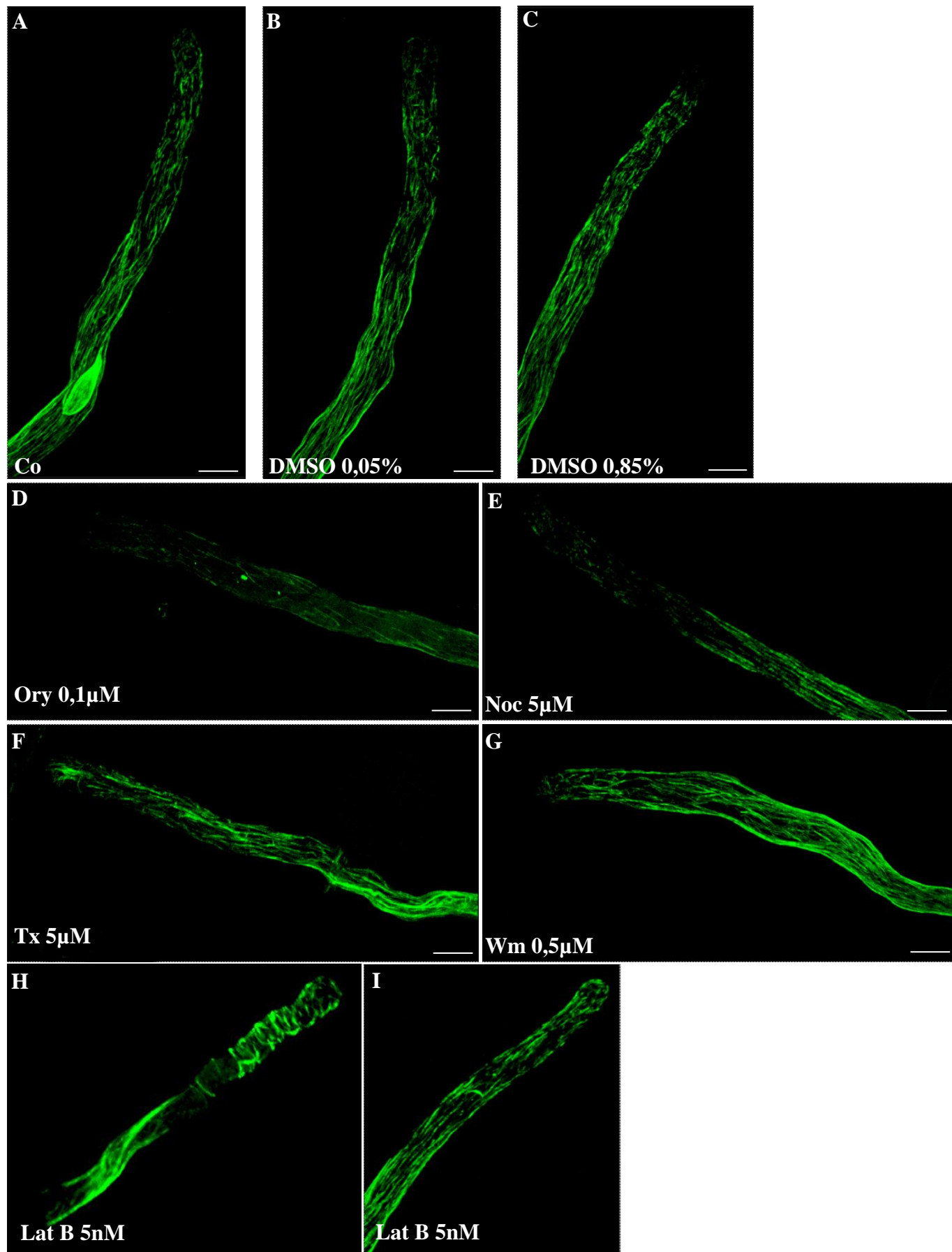

Figure S7

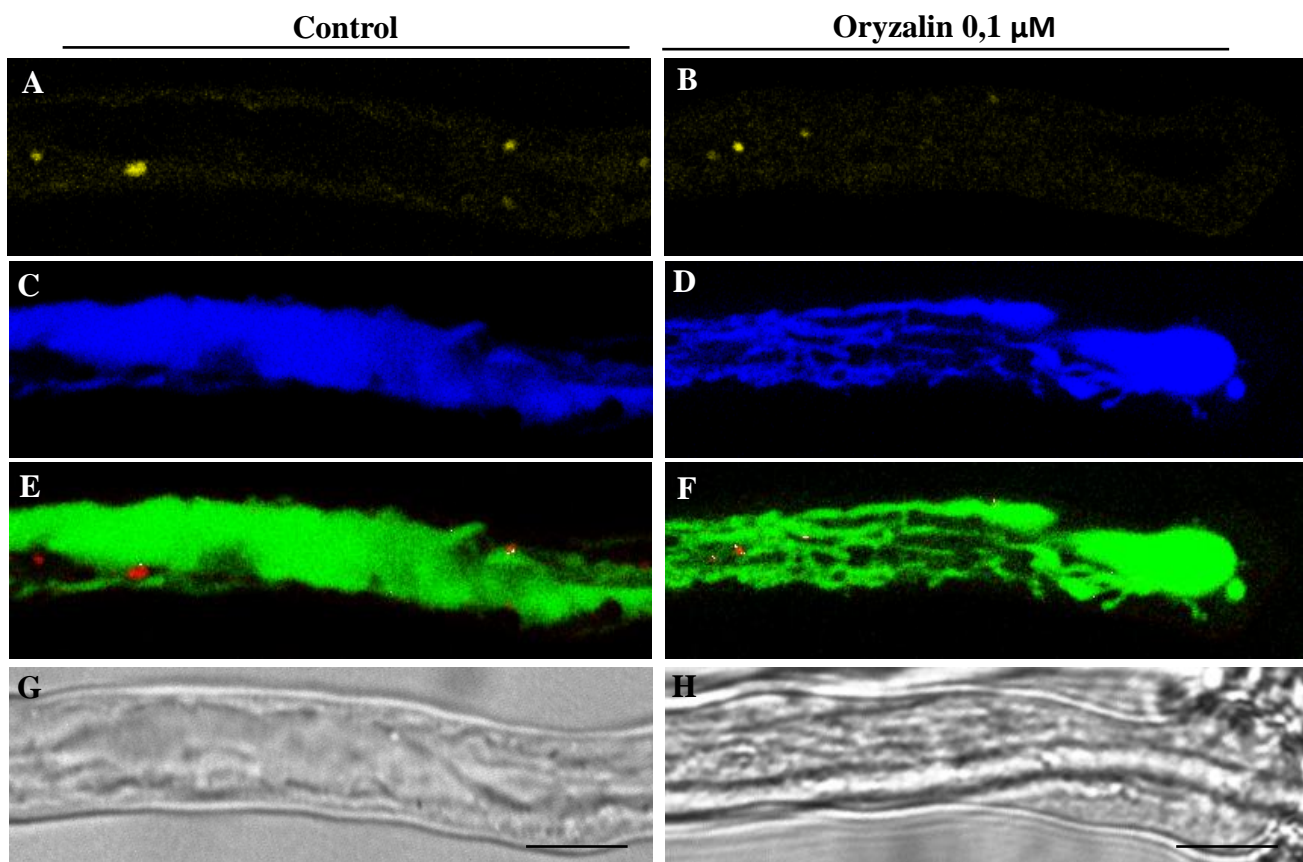

**Fig. S8**

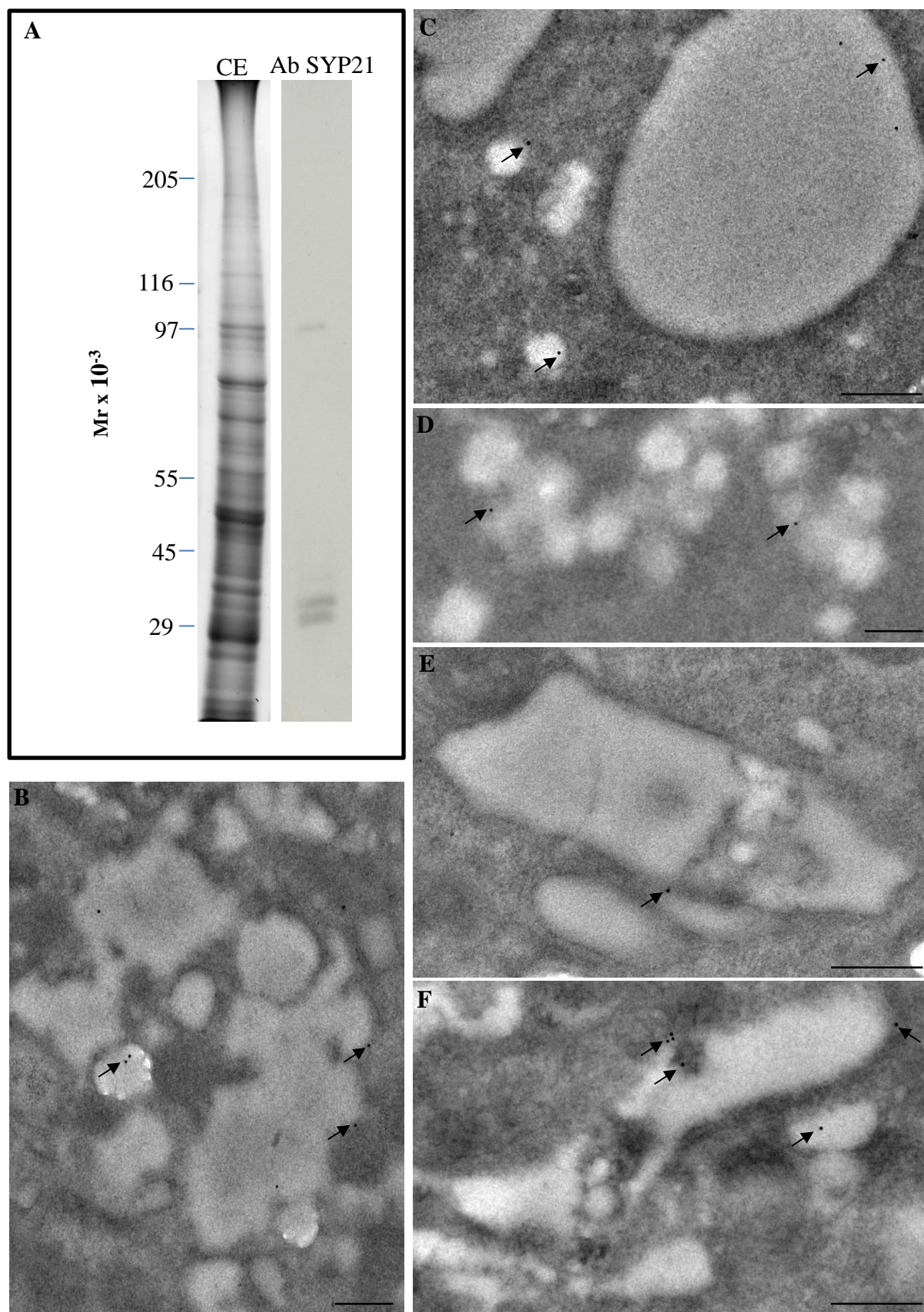

**Figure S9**

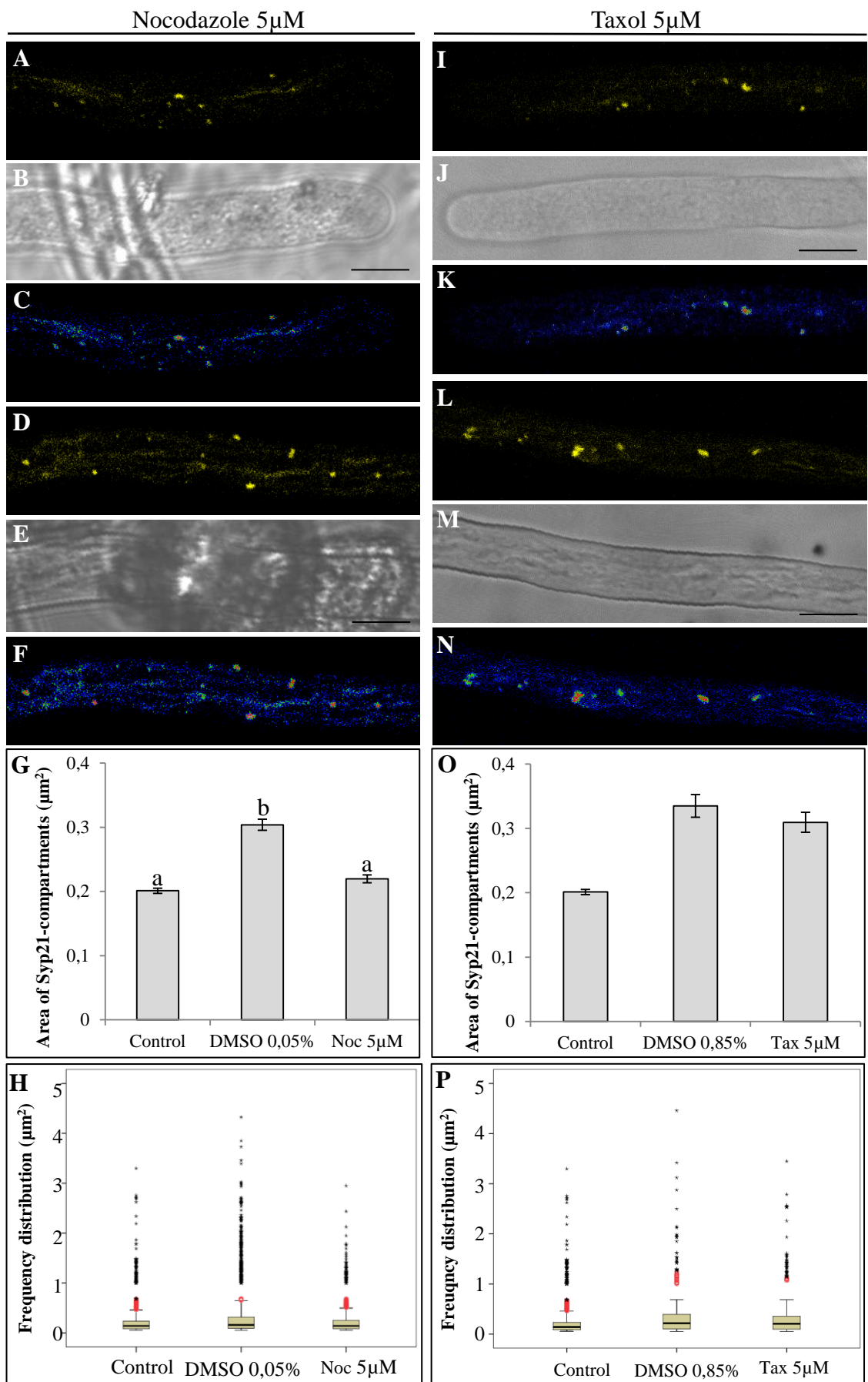

Figure S10

Supplement: Supplementary figures [file rsob180078supp1.pdf]
